# The Balance Effect of π–π Electronic Coupling on NIR‐II Emission and Photodynamic Properties of Highly Hydrophobic Conjugated Photosensitizers

**DOI:** 10.1002/advs.202307569

**Published:** 2023-12-28

**Authors:** Yulin Zhu, Hanjian Lai, Ying Gu, Zixiang Wei, Lin Chen, Xue Lai, Liang Han, Pu Tan, Mingrui Pu, Fan Xiao, Feng He, Leilei Tian

**Affiliations:** ^1^ Shenzhen Grubbs Institute and Department of Chemistry Southern University of Science and Technology Shenzhen 518055 China; ^2^ Department of Materials Science and Engineering Southern University of Science and Technology Shenzhen 518055 China; ^3^ School of Chemistry and Chemical Engineering Harbin Institute of Technology Harbin 150001 China

**Keywords:** π–π interaction, hydrogen bond, NIR‐II fluorescence imaging, organic π‐conjugated molecules, photodynamic therapy

## Abstract

Deep NIR organic phototheranostic molecules generally have large π‐conjugation structures and show highly hydrophobic properties, thus, forming strong π–π stacking in the aqueous medium, which will affect the phototheranostic performance. Herein, an end‐group strategy is developed to lift the performance of NIR‐II emitting photosensitizers. Extensive characterizations reveal that the hydrogen‐bonding interactions of the hydroxyl end group can induce a more intense π–π electronic coupling than the chlorination‐mediated intermolecular forces. The results disclose that π–π stacking will lower fluorescence quantum yield but significantly benefit the photodynamic therapy (PDT) efficiency. Accordingly, an asymmetrically substituted derivative (BTIC‐*δ*OH‐2Cl) is developed, which shows balanced phototheranostic properties with excellent PDT efficiency (14.6 folds of ICG) and high NIR‐II fluorescence yield (2.27%). It proves the validity of the end‐group strategy on controlling the π–π interactions and rational tuning the performance of NIR‐II organic phototheranostic agents.

## Graphical abstract

The hydrogen‐bonding interactions of the hydroxy end group can induce a more intensive π–π electron coupling than the chlorination‐mediated intermolecular forces. π–π stacking will lower fluorescence quantum yield but significantly benefit the PDT efficiency. Therefore, by this end‐group strategy, the phototheranostic properties of the photosensitizers are finely tuned, and a more effective NIR‐II fluorescence‐guided PDT is accomplished.

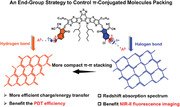

## 1 Introduction

Organic π‐conjugated molecules with strong absorptivity, rich energy level structures, and tunable excited state processes have been widely applied in cancer photo‐theranostics.^[^ ^1^, ^2^, ^3^, ^4^, ^5^, ^6^, ^7^ ^]^ In order to realize deep tissue penetration, π‐conjugated molecules are desired to show NIR absorption and NIR‐II emission, which is gained by extending the π‐conjugation length or constructing electron‐donating‐accepting structures.^[^ ^8^, ^9^, ^10^, ^11^, ^12^ ^]^ Most NIR molecules with extended π‐conjugated length are highly hydrophobic; consequently, they are generally dispersed into the aqueous phase by the nanoprecipitation method. The hydrophobic forces will drive molecules' aggregation during the process, resulting in π–π interactions.^[^ ^13^, ^14^ ^]^ The electronic coupling caused by the intermolecular π–π stacking will notably change the optoelectronic properties of the resultant water‐dispersed nanoparticles (NPs).^[^ ^15^, ^16^, ^17^ ^]^ Therefore, to improve the photo‐theranostic properties of these highly hydrophobic π‐conjugated molecules, only tailoring the molecular structures is insufficient; studying and controlling the π–π electronic coupling effect in the aggregated state becomes an unavoidable issue.

Some strategies have been developed to manipulate the π‐π interaction of π‐conjugated photo‐theranostic molecules in the water‐dispersible NPs. For instance, face‐to‐face H‐type π–π stacking favors non‐radiative decay and significantly quenches the fluorescence of π‐conjugated molecules in the aggregated state.^[^ ^18^ ^]^ As an innovative and successful example to solve this issue, aggregation‐induced emission type π‐conjugated molecules with non‐planar molecular structures can realize high fluorescence in the aggregated state through weakening the intermolecular π–π interactions.^[^ ^19^ ^]^ In another case, we developed a side chain engineering strategy to control the degree of H‐type π stacking in the NPs' aggregated state, realizing improved photoacoustic imaging and NIR‐II fluorescence.^[^ ^16^ ^]^ However, π–π stacking in the aggregated state doesn't always show unfavorable effects, which can also benefit the phototheranostics in enhancing absorptivity and stabilizing the nanostructure (π–π interactions belong to hydrophobic forces).^[^ ^20^ ^]^ Moreover, some recent investigations indicate that strong π–π interaction can effectively promote the excited‐state energy splitting and substantially decreases the singlet‐triplet energy gap (Δ*E*_ST_).^[^ ^21^ ^]^ It is well known that a smaller Δ*E*_ST_ will enhance the efficiency of PDT.^[^ ^22^ ^]^

Recently, some NIR‐II emitting photosensitizers (PSs) have been developed.^[^ ^5^, ^10^ ^]^ However, despite the deep penetration and high resolution of NIR‐II fluorescent emission that will promote the imaging‐guided PDT technique, these NIR‐II PSs generally show low PDT efficiency due to their long‐wavelength absorption determined by lower input energy. In a PDT process, PSs are excited by light and produce cytotoxic reactive oxygen species (ROS) through energy/electron transfer with O_2_ or H_2_O. Therefore, it is critical to intensify the light harvesting and energy/electron transfer capability of NIR‐II PSs in order to make up for the shortage in energy power of the long‐wavelength light and improve their photodynamic properties. As π–π interaction will benefit the above two properties, we were prompted to develop an end‐group strategy to elevate the PDT efficiency of long‐wavelength PSs by enhancing π–π stacking. According to the reports, halogen substituents^[^ ^23^, ^24^ ^]^ for halogen bonding and hydroxyl substituents^[^ ^25^, ^26^ ^]^ for hydrogen bonding are extensively used to mediate π–π stacking. Li et al. introduced hydroxyl end‐groups to ITIC and observed that the hydrogen bonding was capable of organizing π–π stacking for ordered molecular alignments. On the other hand, the chlorine end‐group and the related halogen bonding have attracted research interest, including ours, which have been demonstrated effective in enhancing π–π interactions in many π‐conjugated systems.

**Scheme 1:**
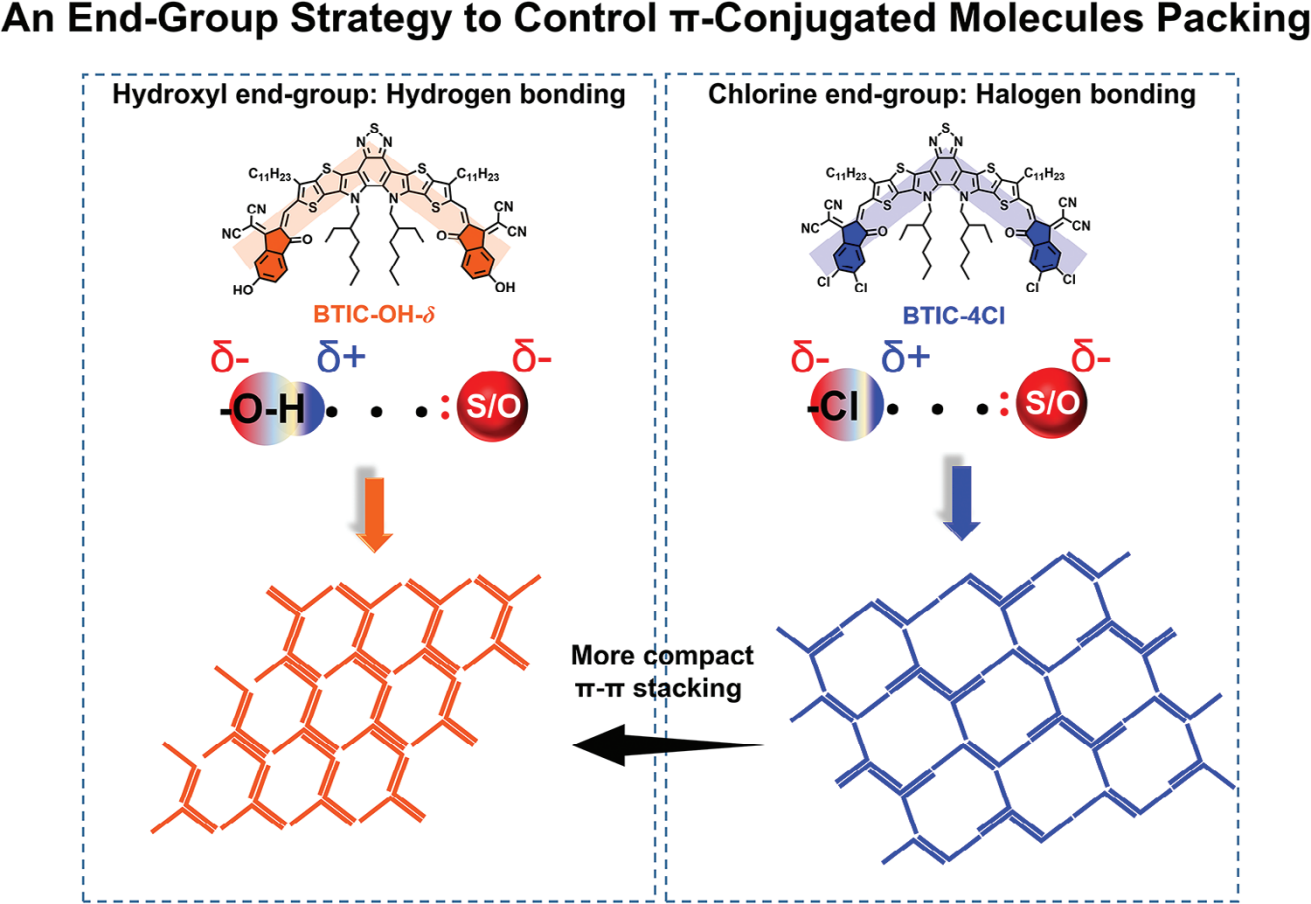
Schematic illustration of an end‐group strategy to control π‐conjugated molecule packing.

Consequently, based on an π‐conjugated molecule, BTIC, four chorine groups (BTIC‐4Cl) and two hydroxy groups (BTIC‐OH‐*δ*) were introduced to explore the control effect of end groups on π–π interactions in this work (**Scheme** **1**). We observed that the hydrogen‐bonding interactions in BTIC‐OH‐*δ* induced a more intense π–π electron coupling than the chlorine‐mediated interactions in BTIC‐4Cl, which was confirmed by single crystal X‐ray diffraction analysis. As a result of the different π–π stacks driven by the end‐group interactions, the absorptivity of BTIC‐OH‐*δ* NPs became 1.17 folds higher than BTIC‐4Cl NPs. In contrast, the absorptivity of BTIC‐OH‐*δ* in the single molecular state was 0.55 folds that of the BTIC‐4Cl molecule. Moreover, the charge mobility of BTIC‐OH‐*δ* in the aggregated states was over 4 folds higher than BTIC‐4Cl. Finally, the in vitro characterization results revealed that the ROS yield of BTIC‐OH‐*δ* NPs was ≈eightfolds higher than BTIC‐4Cl NPs. These results demonstrated that the end‐group strategy could efficiently affect π–π interaction and regulate the PDT properties of highly hydrophobic π‐conjugated molecules in the aggregated state. On the other hand, the π–π packing was beneficial for PDT properties but unfavorable for fluorescence efficiency. Due to such conflicting effects, we designed and synthesized the asymmetrically substituted derivative, BTIC‐*δ*OH‐2Cl, which showed balanced photo‐theranostic properties. BTIC‐*δ*OH‐2Cl exhibited a PDT efficiency comparable to Chlorin e6 (Ce6) and a high NIR‐II fluorescence quantum yield of 2.27%. The cellular and in vivo experiments proved that BTIC‐*δ*OH‐2Cl NPs could realize high‐resolution NIR‐II fluorescence imaging and effective PDT effect to inhibit tumor growth. Overall, the balance effect of π–π electronic coupling on NIR‐II emission and photodynamic properties of π‐conjugated molecules, which was declared and discussed in this work, will provide new ideas for the designs of NIR‐II emitting PSs in the future.

## 2 Results and Discussion

**Scheme 2:**
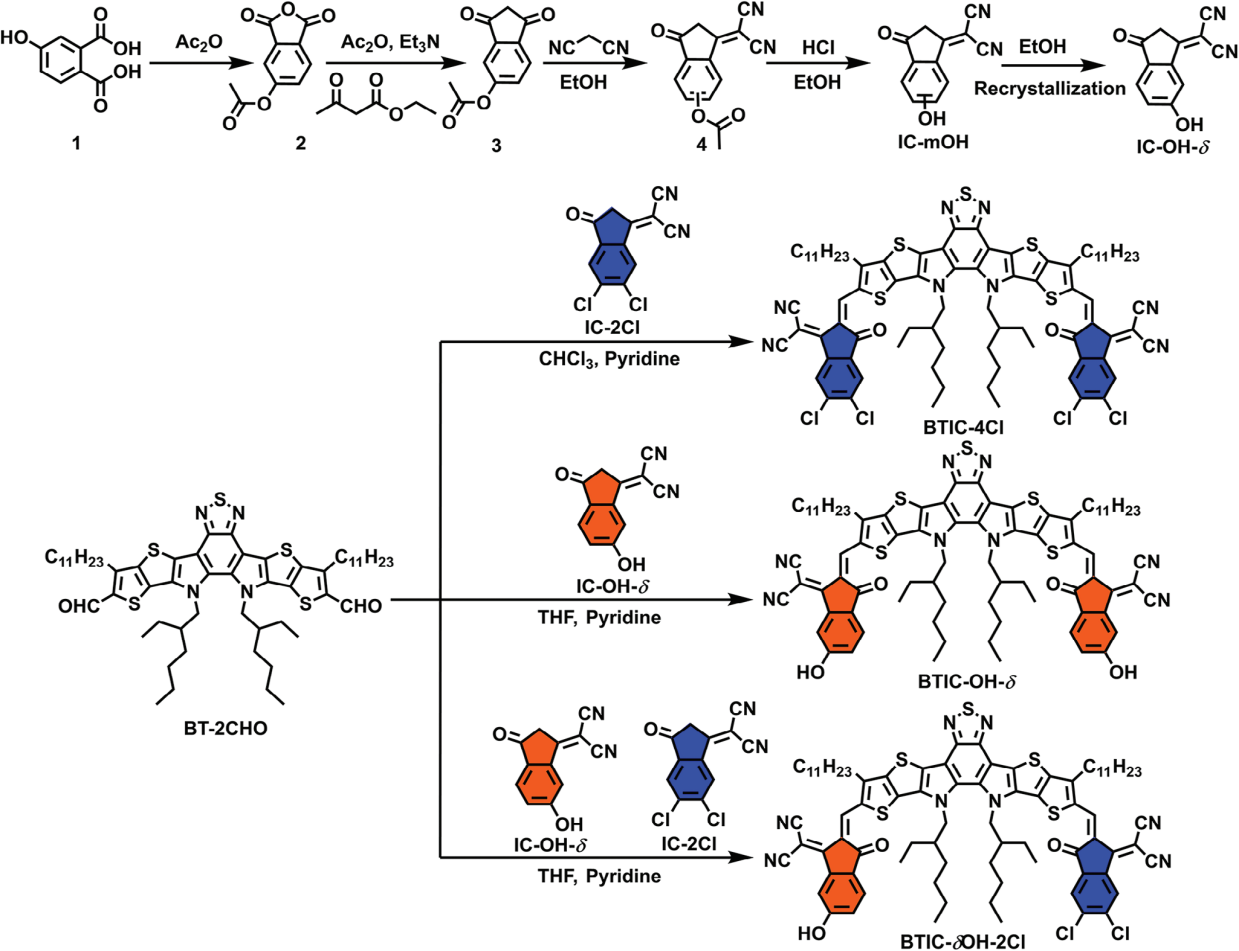
The synthetic routes of IC‐mOH, IC‐OH‐*δ*, BTIC‐4Cl, BTIC‐OH‐*δ*, and BTIC‐*δ*OH‐2Cl.

The chemical structures and synthetic routes for the BTIC‐4Cl, BTIC‐OH‐*δ*, and BTIC‐*δ*OH‐2Cl are shown in **Scheme** **2**. They are all A–D–A) π‐conjugated structures comprising benzo[1,2‐b:4,5‐b’]dithiophene (BT) 7‐ring fused core and chloride (‐Cl), or hydroxyl (‐OH) substituted 1,1‐dicyanomethylene‐3‐indanone (IC) ends. IC‐OH was synthesized in four steps from 4‐hydroxyphthalic acid (1) and purified by recrystallization from ethanol. BTIC‐4Cl and BTIC‐OH‐*δ* were synthesized through Knoevenagel condensation reactions^[^ ^27^ ^]^ of IC‐2Cl or IC‐OH‐*δ* with dialdehyde BT‐2CHO. Meanwhile, the asymmetrical BTIC‐*δ*OH‐2Cl was obtained by controlling the feed ratio of IC‐2Cl and IC‐OH, purified by careful purification via high‐performance liquid chromatography. For the three target molecules, ─Cl and ─OH substituents can form halogen bonding and hydrogen bonding and are supposed to affect intermolecular packing and π–π stacking. Notably, due to the strong intermolecular interactions, a small amount of tetrahydrofuran was needed to break hydrogen bonds and assist BTIC‐OH‐*δ* and BTIC‐*δ*OH‐2Cl in being dissolved in most organic solvents. The synthesis procedures and structural characterization are detailed in Figures S1–S8 (Supporting Information). The presence of hydrogen bonding interactions through the hydroxyl substitution was proved by infrared spectroscopy (Figure S9, Supporting Information).

**Figure 1:**
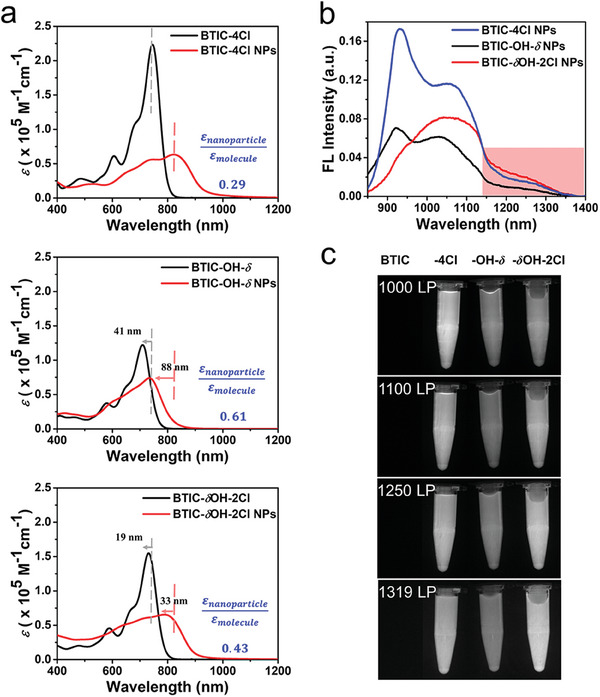
a) Absorption spectra of BTIC‐4Cl, BTIC‐OH‐*δ*, and BTIC‐*δ*OH‐2Cl in chloroform solution and NPs dispersed in water. Fluorescence spectra (b) and NIR‐II fluorescence images (c) of BTIC‐4Cl, BTIC‐OH‐*δ*, and BTIC‐*δ*OH‐2Cl NPs dispersed in water under 808 nm laser irradiation (with equivalent absorbance at 808 nm).

We first characterized the monodisperse properties of BTIC‐4Cl, BTIC‐OH‐*δ*, and BTIC‐*δ*OH‐2Cl. At the B3LYP/631G(d,p) level, the charge separation properties of BTIC‐4Cl, BTIC‐OH‐*δ*, and BTIC‐*δ*OH‐2Cl were studied using Gaussian 09 density functional theory. Side chains on the core unit were replaced by methyl to simplify the calculation (Figure S10, Supporting Information). The results showed that the electron density of the highest occupied molecular orbital was mainly delocalized along the conjugated backbones, and the lowest unoccupied molecular orbital was primarily localized on the electron‐deficient subunits, indicating that the D‐A electronic structures in the three molecules were not affected by the ─Cl/─OH substituents. Further, UV–vis absorption spectra showed absorption peaks at 750 nm (BTIC‐4Cl), 709 nm (BTIC‐OH‐*δ*), and 731 nm (BTIC‐*δ*OH‐2Cl), respectively (**Figure** **1**a). Additionally, the corresponding molar extinction coefficients were 2.20 × 10^5^ (BTIC‐4Cl), 1.22 × 10^5^ (BTIC‐OH‐*δ*), and 1.55 × 10^5^ L mol^−1^ cm^−1^ (BTIC‐*δ*OH‐2Cl) (**Table** **1**). These results revealed that substituents changed the D–A strengths in those molecules. The more electron‐deficient ─Cl substitution enhanced the A–D strength, resulting in a narrower bandgap, more intense NIR absorption, and higher absorption coefficient. On the contrary, ─OH resulted in relatively weaker A–D strength in BTIC‐OH‐*δ*. As expected, BTIC‐*δ*OH‐2Cl with both ─Cl and ─OH substitutions showed properties intermediating between BTIC‐4Cl and BTIC‐OH‐*δ*.

**Table 1:** The basic photophysical parameters of BTIC‐4Cl, BTIC‐OH‐*δ*, and BTIC‐*δ*OH‐2Cl.

	Solutions^a)^		Aggregates in nanoparticles^b)^				
	λ_abs_ [nm]	ε 10^5^ [L mol^−1^·cm^−1^]	λ_abs_ [nm]	ε 10^5^ [L mol^−1^·cm^−1^]	Δλ_abs_ [nm]	PLQY [%]^c)^	ROS Yield [vs.ICG]
**BTIC‐4Cl**	750	2.20	823	0.63	73	3.76	3.3
**BTIC‐OH‐δ**	709	1.22	735	0.74	26	1.95	25.9
**BTIC‐δOH‐2Cl**	731	1.55	790	0.66	59	2.27	14.6

^a)^ BTIC‐4Cl, BTIC‐OH‐*δ*, or BTIC‐*δ*OH‐2Cl solution in CHCl_3_;

^b)^ BTIC‐4Cl, BTIC‐OH‐*δ*, or BTIC‐*δ*OH‐2Cl NPs in water;

^c)^ IR‐26 dye as a reference (PLQY = 0.5%).^[^ ^35^ ^]^

Subsequently, the hydrophobic BTIC‐4Cl, BTIC‐OH‐*δ*, and BTIC‐*δ*OH‐2Cl were encapsulated into an amphiphilic block copolymer, 1,2‐stearoyl‐sn‐glycerol‐3‐phosphoethanolamine‐N‐[hydroxyl(poly (ethylene glycol))−2000] (DSPE‐PEG_2000_) by nanoprecipitation method to form nanoparticle in aqueous media. According to transmission electron microscopy (TEM) and dynamic light scattering (DLS) analysis, the three NPs exhibit similar morphologies and size distributions. TEM micrographs (Figure S11, Supporting Information) showed the uniform spherical morphology of BTIC‐4Cl, BTIC‐OH‐*δ*, and BTIC‐*δ*OH‐2Cl NPs with diameters ≈35, 40, and 38 nm. Consistently, hydrodynamic diameters of BTIC‐4Cl, BTIC‐OH‐*δ*, and BTIC‐*δ*OH‐2Cl NPs in ultra‐pure water were ≈45, 50, and 49 nm according to DLS (Figure S12, Supporting Information). The sizes of NPs were suitable to enhance the permeability and retention effect for passive tumor delivery.^[^ ^28^, ^29^ ^]^ The element mapping was performed to evaluate the element distribution within NPs, which confirmed the uniform distributions of BTIC‐4Cl, BTIC‐OH‐*δ*, and BTIC‐*δ*OH‐2Cl in NPs (Figure S13, Supporting Information). The zeta potentials of BTIC‐4Cl, BTIC‐OH‐*δ*, and BTIC‐*δ*OH‐2Cl NPs in ultra‐pure water showed weak electronegativity (Figure S14, Supporting Information) due to the surface‐bound PEG. Moreover, these NPs showed good long‐term colloidal stability in water (Figure S15, Supporting Information).

Compared with the monodispersed state, the aggregated state of NPs showed red‐shifted and broader absorption bands, suggesting the co‐existence of various π–π stacking structures.^[^ ^30^, ^31^ ^]^ The absorption peaks of BTIC‐4Cl, BTIC‐OH‐*δ*, and BTIC‐*δ*OH‐2Cl NPs shifted to 823, 735, and 790 nm, respectively (Figure 1a). As a result of polymeric encapsulation and attenuation, their molar extinction coefficients decreased to 6.33 × 10^4^ (BTIC‐4Cl), 7.39 × 10^4^ (BTIC‐OH‐*δ*), and 6.55 × 10^4^ L mol^−1^ cm^−1^ (BTIC‐*δ*OH‐2Cl) (Table 1). Among these three molecules, BTIC‐OH‐*δ* showed the biggest absorption coefficient in the aggregated nanoparticle state, whereas it was the smallest one in the monodispersed state. The *ε*_nanoparticle_ */ε*_molecule_ values were determined to be 0.29, 0.61, and 0.43 for BTIC‐4Cl, BTIC‐OH‐*δ*, and BTIC‐*δ*OH‐2Cl, respectively. The results implied that a more intense intermolecular π–π electronic coupling might be formed in BTIC‐OH‐*δ* NPs than in the other two NPs. In addition, the three NPs also showed different degrees of red‐shift in absorption relative to the solutions, i.e., 73 nm for BTIC‐4Cl, 26 nm for BTIC‐OH‐*δ*, and 59 nm for BTIC‐*δ*OH‐2Cl (Table 1). Relative to BTIC‐4Cl, BTIC‐OH‐*δ* showed a more significant blue shift in the aggregated state (88 nm) in comparison with the monodispersed state (41 nm). Therefore, taking the above information together, we hypothesized that H‐aggregates were predominated in BTIC‐OH‐*δ* NP as it exhibited intense π–π interactions and more blue‐shifted absorption. Generally, intermolecular interaction‐induced π–π stacks may cause two different effects: 1) the excitonic coupling effect depending on intermolecular π electronic coupling.^[^ ^32^, ^33^ ^]^ The closer π–π distance and larger π electronic overlap will lead to a more red‐shifted absorption in the aggregated state. 2) the coulombic coupling effect relying on the geometry of the π–π stacking.^[^ ^34^ ^]^ Specifically, the excitation energy of the “head‐to‐tail” J‐aggregates is smaller than the monomers, resulting in a red‐shifted absorption; on the contrary, the excitation energy of the “face‐to‐face” H‐aggregates is larger than the monomers, resulting in a blue‐shifted absorption. Accordingly, we observed that all three NPs red‐shifted compared with their mono‐dispersed state as a result of the excitonic coupling effect. However, if H‐aggregates are more predominant in BTIC‐OH‐*δ* NP than the other two NPs, the great blue shift of BTIC‐OH‐*δ* among the three aggregated states can be well explained.

**Figure 2:**
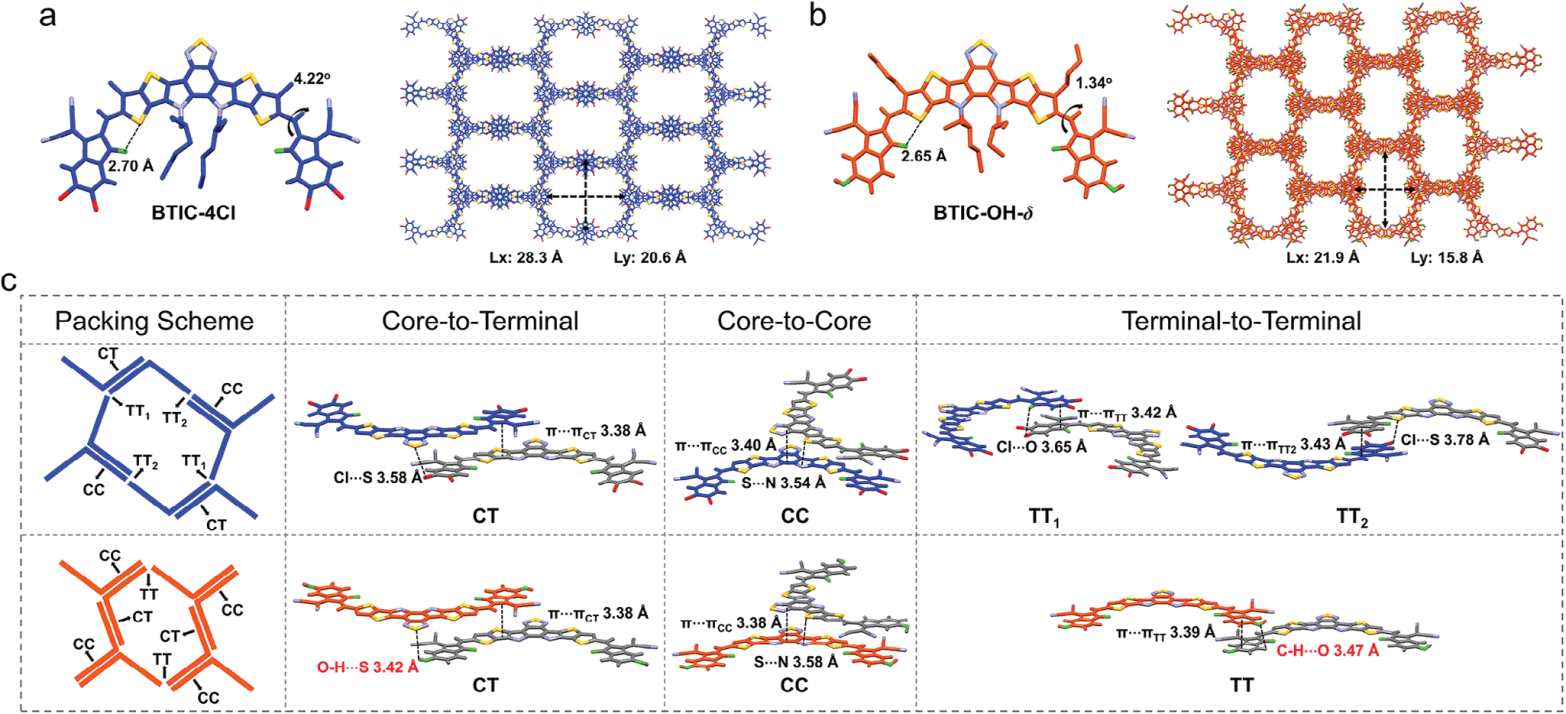
The single‐crystal structures of a) BTIC‐4Cl and b) BTIC‐OH‐*δ*. c) The molecular packing of BTIC‐4Cl (blue) and BTIC‐OH‐*δ* (red) by single‐crystal data analysis.

In order to prove our deductions in the molecular packing of this series of molecules in the aggregated states, their single crystals were cultured and analyzed (**Figure** **2**; Figure S16, Supporting Information). Due to the S∙∙∙O interlock effect, both BTIC‐4Cl and BTIC‐OH‐*δ* showed relatively planar molecular conformation. However, the dihedral angle between the planes of the ring‐fused core and IC end group of BTIC‐4Cl (4.22^o^) is larger than that of BTIC‐OH‐*δ* (1.34^o^), resulting from the steric hindrance induced by the large size of the chlorine substituents. Consistently, the more planar conformation of BTIC‐OH‐*δ* will facilitate the formation of H‐aggregation. Chloride substituent and its mediated halogen interactions have recently been widely investigated in organic semiconducting materials. The σ^*^ anti‐bonding orbital is likely to form orbital–orbital interaction with the lone pair orbital of S and O.^[^ ^36^ ^]^ In the crystal structure of BTIC‐4Cl, Cl···S and Cl···O interactions were found to play a crucial role in organizing π–π stacking. On the other hand, hydroxyl substituent in BTIC‐OH‐*δ* will mediate the hydrogen bonding interaction that is present between an H atom and an electronegative atom, such as F, N, O, and S. Due to the steric hindrance, no direct O‐H···O hydrogen bonds formed among hydroxyl groups. However, well‐defined O‐H···S and C‐H···O hydrogen bonds were observed in molecular packing structures. With halogen bonding and hydrogen bonding, both BTIC‐4Cl and BTIC‐OH‐*δ* formed 3D network molecular stacking, and each frame of the 3D network was formed by the π–π stacks of eight molecules. However, their molecular packing modes were different, resulting in different frame sizes. There are four stacking modes in the BTIC‐4Cl single‐crystal structure: core‐to‐terminal (CT) aggregation, core‐to‐core (CC) aggregation, and two types of terminal‐to‐terminal (TT) aggregation. For the BTIC‐OH‐*δ* single‐crystal structure, there is only one type of terminal‐to‐terminal aggregation. Each frame in the network of BTIC‐4Cl single‐crystal comprised 2 CT aggregations, 2 CC aggregations, 2 TT_1_ aggregations, and 2 TT_2_ aggregations. In comparison, the frame of BTIC‐OH‐*δ* had 2 CT aggregations, 4 CC aggregations, and 2 TT aggregations. Therefore, due to more core‐to‐core aggregations, a much smaller frame size was formed from BTIC‐OH‐*δ* (21.9 Å × 15.8 Å) in comparison with BTIC‐4Cl (28.3 Å × 20.6 Å). A smaller frame size can represent an intenser π–π stacking. Moreover, the core‐to‐core and core‐to‐terminal aggregations show larger intermolecular π‐electronic overlap and behave more similarly to H‐aggregation. Accordingly, we discovered that the crystal structures could well coincide with the above speculations, i.e., BTIC‐OH‐*δ* NPs with the predominance of H‐aggregation showed a more intense π–π coupling. We hypothesized that the large size of the chloride atom would induce more significant steric hindrance; For the asymmetric molecular BTIC‐*δ*OH‐2Cl, we failed in culturing its single crystal after many attempts. However, the properties of BTIC‐*δ*OH‐2Cl were intermediate between BTIC‐OH‐*δ* and BTIC‐4Cl, which can be well explained by comprehensively considering the other two materials.

As H‐aggregation is typically none‐emissive and serves as a non‐radiative decay channel, the fluorescence properties of the NPs were investigated.^[^ ^37^ ^]^ The NIR‐II fluorescence spectra of BTIC‐4Cl, BTIC‐OH‐*δ*, and BTIC‐*δ*OH‐2Cl NPs (their absorbances at 808 nm were equivalent) ranged from 850 to 1400 nm under an 808 nm laser excitation (Figure 1b). Fluorescence quantum yields (850–1400 nm) were determined to be 3.76% (BTIC‐4Cl), 1.95% (BTIC‐OH‐*δ*), and 2.27% (BTIC‐*δ*OH‐2Cl), respectively (Figure S17, Supporting Information, relative to the fluorescence quantum yield of IR‐26 of 0.50% in dichloroethane^[^ ^35^ ^]^). These results coincide with the above deduction that BTIC‐OH‐*δ* NPs with more H‐aggregated π–π stacking showed the lowest fluorescence quantum yield. According to the overall fluorescence quantum yields, BTIC‐4Cl NP is the most efficient emitter, and BTIC‐*δ*OH‐2Cl NP is the intermediate one. However, BTIC‐*δ*OH‐2Cl NPs showed slightly red‐shifted emission, resulting in higher fluorescence at the long wavelength region. Therefore, by comparing the digital photos of these NPs (with equivalent absorbance at 808 nm) under the different long‐pass (LP) filters, it can be clearly seen that BTIC‐4Cl NPs emitted the brightest NIR‐II fluorescence under 1000 LP and 1100 LP. Interestingly, under the deeper 1250 LP and 1319 LP, BTIC‐*δ*OH‐2Cl NPs emitted a brighter NIR‐II fluorescence than BTIC‐4Cl NPs due to the slightly red‐shifted emission of BTIC‐*δ*OH‐2Cl NPs (Figure 1c).

**Figure 3:**
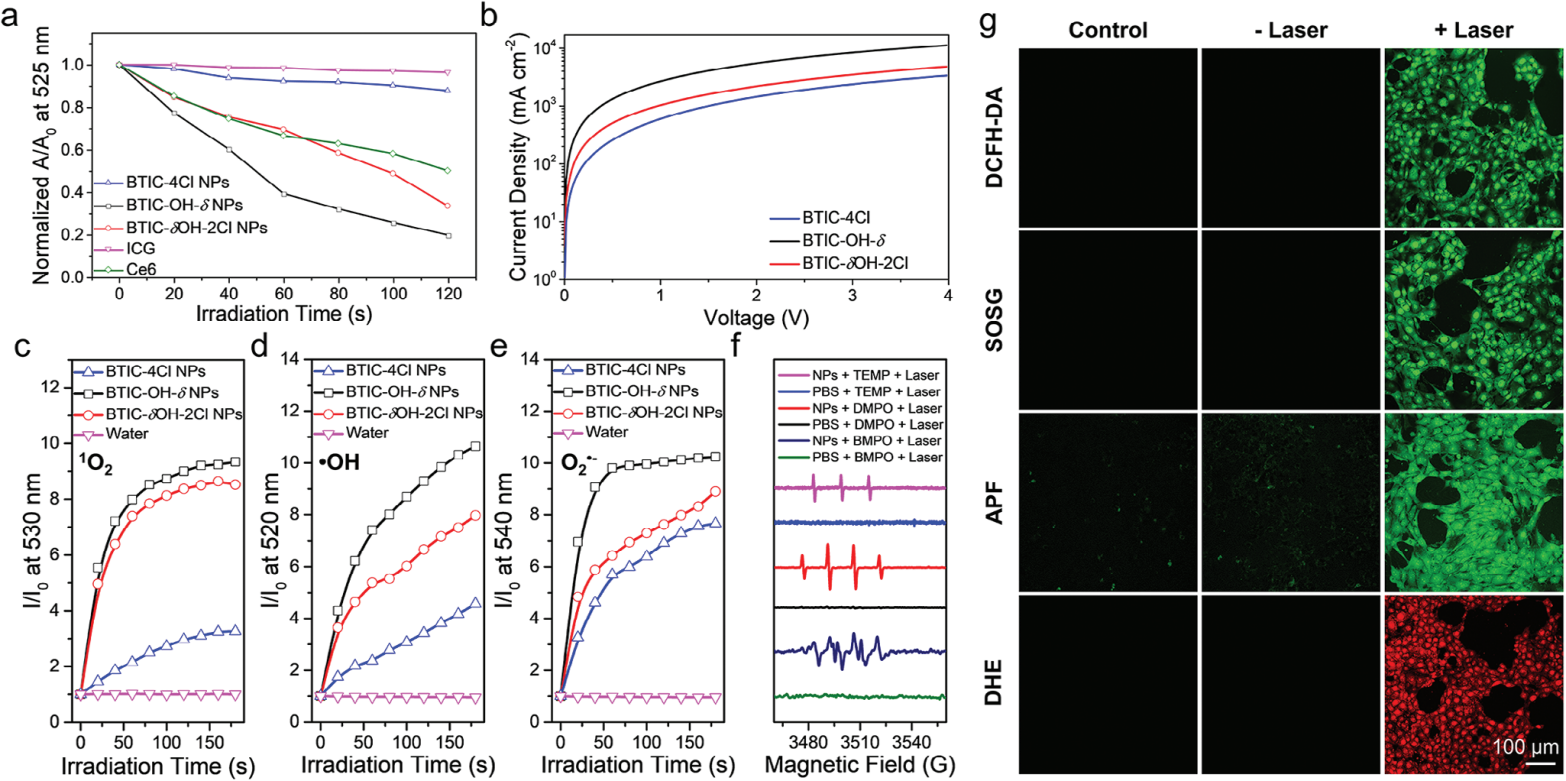
a) Plots of decomposition rates of DPBF (for general ROS detection) in the presence of BTIC‐4Cl, BTIC‐OH‐*δ*, BTIC‐*δ*OH‐2Cl, and Ce6 NPs, or ICG (with equivalent absorbance at 808 or 660 nm) in water along the irradiation time (808 or 660 nm laser, 0.6 W cm^−2^). b) The electron mobility curves of BTIC‐OH‐*δ*, BTIC‐*δ*OH‐2Cl, and BTIC‐4Cl in film. Plots of relative PL intensity of SOSG c) (for ^1^O_2_ detection), APF d) (for •OH detection), and DHR 123 e) (for O_2_^•−^ detection) in the presence of BTIC‐4Cl, BTIC‐OH‐*δ*, and BTIC‐*δ*OH‐2Cl NPs, or ICG (with equivalent absorbance at 808 nm) in water along the irradiation time (808 nm laser, 0.6 W cm^−2^). f) EPR spectra of TEMP/^1^O_2_, DMPO/•OH, and BMPO/O_2_^•−^ for BTIC‐*δ*OH‐2Cl NPs under 808 nm irradiation at 1.0 W cm^−2^ for 10 min. g) ROS detection in 4T1 cells using DCFH‐DA, SOSG, AFP, and DHE as total ROS, ^1^O_2_, •OH, and O_2_^•−^ fluorescence indicator, respectively. NIR light irradiation (808 nm, 0.6 W cm^−2^, 10 min) was conducted after cells were incubated with BTIC‐*δ*OH‐2Cl NPs (25 µg mL^−1^). A_0_ and A represent the absorbances of DPBF before and after irradiation, respectively. I_0_ and I represent the PL intensities of the indicator before and after irradiation, respectively.

In addition to optical properties, π–π stacking also determines the energy and electron transfer in the aggregated state, which plays a crucial role in photodynamic reactions.^[^ ^33^ ^]^ Thus, the PDT properties of BTIC‐4Cl, BTIC‐OH‐*δ*, and BTIC‐*δ*OH‐2Cl NPs were studied by comparing their ROS generation efficiency. The ROS generation efficiency of BTIC‐4Cl, BTIC‐OH‐*δ*, and BTIC‐*δ*OH‐2Cl NPs were determined by comparing them with the commercially available photosensitizers, Ce6, and ICG. First, 1,3‐diphenylisobenzofurane (DPBF) was used as a total ROS indicator. Its absorption intensity will decrease with the production of ROS species. As Ce6 is a hydrophobic molecule, Ce6 NPs were fabricated by a similar nanoprecipitation method. **Figure** **3**a and Figure S18 (Supporting Information) showed that the absorption intensity of DPBF continuously decreased under 808 nm laser irradiation in the presence of BTIC‐4Cl, BTIC‐OH‐*δ*, and BTIC‐*δ*OH‐2Cl NPs. After laser irradiation for 120 s, the ROS yields of BTIC‐4Cl, BTIC‐OH‐*δ*, BTIC‐*δ*OH‐2Cl NPs, and Ce6 were determined to be 3.3, 25.9, 14.6, and 13.9 folds of ICG, respectively (Figure S18b, Supporting Information).^[^ ^5^ ^]^ Surprisingly, the ROS yield of BTIC‐OH‐*δ* was ≈eightfolds higher than that of BTIC‐4Cl. If considering at the single‐molecular level, the more electron‐deficient ─Cl substitution enhanced the A–D strength in BTIC‐4Cl. Generally, a strong intramolecular charge transfer effect would reduce the energy gap between the singlet and triplet excited states (∆*E*_ST_) and promote the PDT‐related intersystem crossing process (k_ISC_).^[^ ^38^, ^39^ ^]^ However, the PDT performance of BTIC‐4Cl NPs was not up to BTIC‐OH‐*δ* NPs, which may arise from the different molecular packing in the aggregated state.^[^ ^40^ ^]^ The space charge limited current (SCLC) method was utilized to test the charge mobility of BTIC‐4Cl, BTIC‐OH‐*δ*, and BTIC‐*δ*OH‐2Cl in films (aggregated states), revealing the results of 1.3 × 10^−4^, 5.6 × 10^−4^, and 2.2 × 10^−4^ mA cm^−2^, respectively (Figure 3b). This experiment directly demonstrated that the charge transport capability of BTIC‐OH‐*δ* was ≈fourfolds higher than BTIC‐4Cl in the aggregated state. Therefore, it clarifies that the more intense π–π electronic coupling in BTIC‐OH‐*δ* NPs indeed would promote charge/energy transfer, which will further improve the PDT photochemical reactions.

Further, their Type II and Type I PDT properties were studied by distinct probes, singlet‐oxygen‐sensor‐green (SOSG), aminophenyl fluorescein (APF), and dihydrorhodamine 123 (DHR123), which are non‐fluorescent but can emit strong fluorescence when reacting with ^1^O_2_, •OH and O_2_^•−^, respectively. As depicted in Figure 3c**–**e and Figures S19–S21 (Supporting Information), under 808 nm laser irradiation for 180 s, the emission of SOSG at 530 nm (detecting ^1^O_2_, the product of Type II PDT) increased by 3.3, 9.3, 8.5, and 1.1 folds for BTIC‐4Cl NPs, BTIC‐OH‐*δ* NPs, BTIC‐*δ*OH‐2Cl NPs, and ICG (with equivalent absorbance at 808 nm). The emission of APF at 520 nm (detecting •OH, the product of Type I PDT) increased by 4.6, 10.6, 8.0, and 1.2 folds for BTIC‐4Cl NPs, BTIC‐OH‐*δ* NPs, BTIC‐*δ*OH‐2Cl NPs, and ICG (with equivalent absorbance at 808 nm). The emission of DHR123 at 540 nm (detecting O_2_^•−^, the product of Type I PDT) increased by 7.7, 10.2, and 8.9 folds for BTIC‐4Cl NPs, BTIC‐OH‐*δ* NPs, and BTIC‐*δ*OH‐2Cl NPs (with equivalent absorbance at 808 nm). These results showed that the ROS species produced by BTIC‐4Cl, BTIC‐OH‐*δ*, and BTIC‐*δ*OH‐2Cl NPs were from a combination of Type II and Type I PDT processes.

To explain this mechanism, we first looked at the production of O_2_^•−^ through Type‐I PDT, which occurs when photoexcited electrons react with O_2_. Our results from cyclic voltammetry (CV) experiments (Figure S1, Supporting Information) showed that the reducing potentials of the photoexcited electrons of BTIC‐4Cl (−0.54 V), BTIC‐OH‐*δ* (−0.56 V), and BTIC‐*δ*OH‐2Cl (−0.55 V) were more negative than that of O_2_(−0.33 V vs NHE, pH 7),^[^ ^41^ ^]^ indicating that the electrons could potentially reduce O_2_ to O_2_^•−^. X‐ray photoelectron spectroscopy (XPS) valence band spectra (Figure S23, Supporting Information) were used to determine the oxidizing potentials of the photoexcited holes of BTIC‐4Cl (3.77 V), BTIC‐OH‐*δ* (3.87 V), and BTIC‐*δ*OH‐2Cl (3.82 V) were higher than the oxidation potential of H_2_O (1.99 V vs NHE, pH 7),^[^ ^41^ ^]^ indicating that the photo‐excited holes of BTIC‐4Cl, BTIC‐OH‐*δ*, and BTIC‐*δ*OH‐2Cl NPs have the potential to oxidize H_2_O to •OH. Moreover, BTIC‐OH‐*δ* NPs showed enhanced Type II and Type I PDT efficiency in comparison with BTIC‐4Cl NPs, consistent with the fact that the enhanced π–π stacking would benefit both energy (corresponding to Type II mechanism) and electron transfer (corresponding to Type I mechanism) in aggregated state of these organic semiconducting materials, finally facilitating photochemical reactions between the encapsulated PSs and O_2_ or H_2_O in the environment.

**Figure 4:**
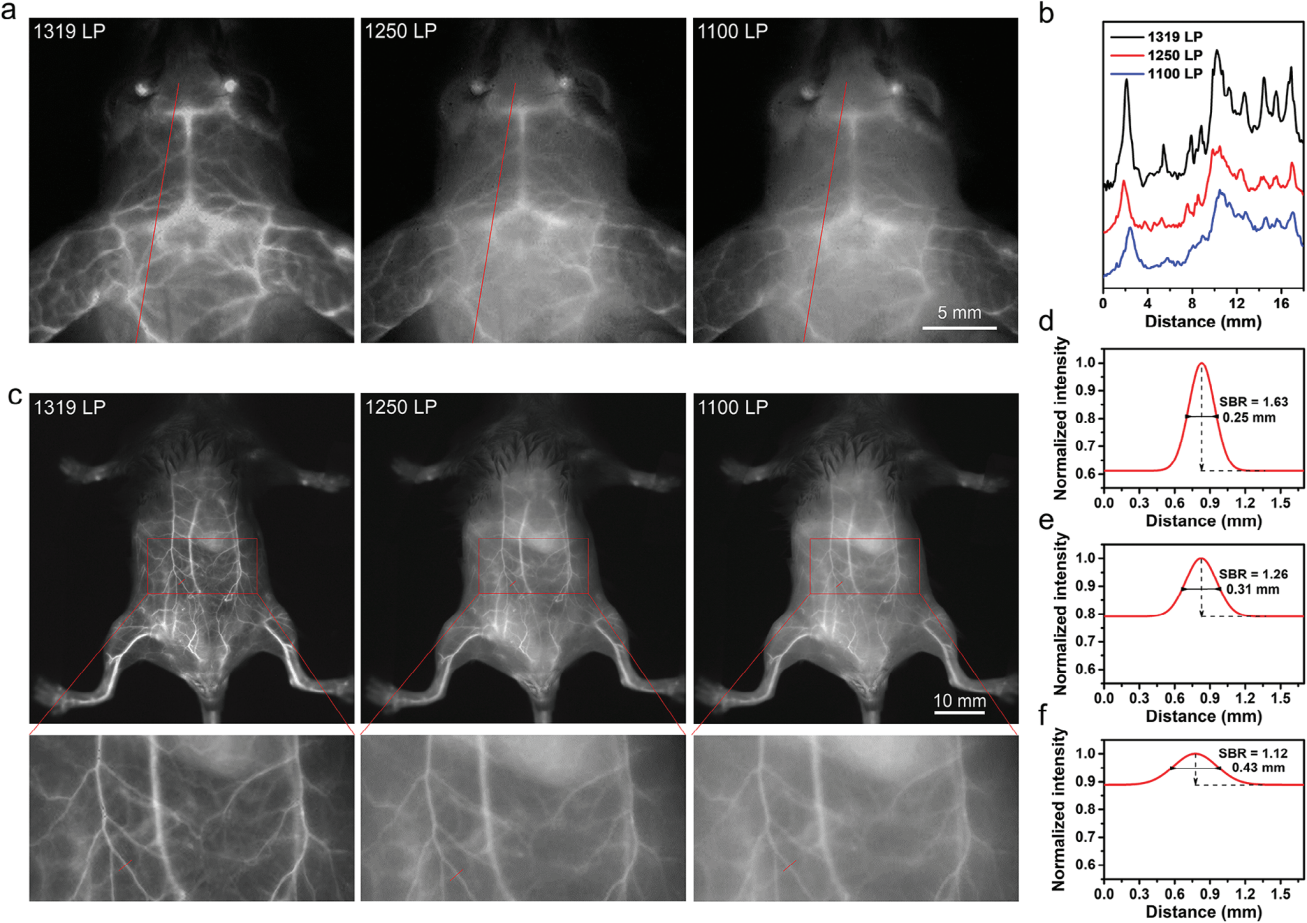
a) Imaging of cerebral vessels and corresponding line cuts. b,c) whole‐body NIR‐II fluorescein angiography, vessel details from corresponding boxes, and line cut analysis d) 1319 LP, e) 1250 LP, and f) 1100 LP after intravenous injection of BTIC‐*δ*OH‐2Cl NPs under different filters.

The intense π–π stacking significantly enhanced the PDT efficiency of BTIC‐OH‐*δ* NPs relative to BTIC‐4Cl NPs, however, which also lowered the NIR‐II fluorescence quantum yield of BTIC‐OH‐*δ* NPs. NIR‐II fluorescence imaging will be very crucial for theranostic applications. BTIC‐*δ*OH‐2Cl NPs with both ─Cl and ─OH substitutions carry properties intermediating between BTIC‐4Cl and BTIC‐OH‐*δ*, which exhibited a PDT efficiency of 14.6 folds of ICG and a high NIR‐II fluorescence quantum yield of 2.27%. Especially under the deeper 1250 LP and 1319 LP, BTIC‐*δ*OH‐2Cl NPs emitted a brighter NIR‐II fluorescence than BTIC‐4Cl NPs (Figure 2c), which will benefit the signal‐to‐noise ratio (SBR) of bioimaging applications. Accordingly, the feasibility of BTIC‐*δ*OH‐2Cl NPs for in vivo imaging application was estimated. After intravenous injection of BTIC‐*δ*OH‐2Cl NPs (0.5 mg mL^−1^, 0.2 mL), images of the whole body and cerebral vessels of healthy mice were obtained under 808 nm laser irradiation (**Figure** **4**). Systemic blood vessels could be clearly observed 10 min after injection of BTIC‐*δ*OH‐2Cl NPs. Fluorescence imaging of abdominal vessels confirmed that the spatial resolution was higher under a longer wavelength LP filter. Quantitative analysis (Figure 4d‐f) showed that the SBR for the images using 1319, 1250, and 1100 nm LP filters were 1.63, 1.26, and 1.12, respectively. Impressively, NIR‐II images using the 1319 and 1250 nm LP filters showed improved contrast by suppressing the background compared to images captured using the 1100 nm filter. More detailed information could be discerned under the 1319 nm LP filter taking the imaging of cerebral vessels as an example (Figure 4a,b).

Considering that BTIC‐*δ*OH‐2Cl NPs showed superior capability in NIR‐II fluorescence imaging, its PDT performance in vitro was evaluated. The production of ^1^O_2_, •OH and O_2_^•−^ by BTIC‐*δ*OH‐2Cl NPs were verified by electron spin resonance (EPR) technique using 2,2,6,6‐tetramethylpiperidine (TEMP), 5,5‐dimethyl‐1‐pyrroline‐N‐oxide (DMPO) and 3,4‐dihydro‐2‐methyl‐1,1‐dimethylethyl ester‐2H‐pyrrole‐2‐carboxylic acid‐1‐oxide (BMPO) as spin trapping agents. As shown in Figure 3f, after 600 J cm^−2^ (808 nm, 1 W cm^−2^ for 10 min) laser irradiation, the characteristic 1:1:1 multiplicity of TEMP/^1^O_2_ adduct, the 1:2:2:1 multiplicity of DMPO/•OH adduct and the hyperfine splits of BMPO/O_2_^•−^ adduct were observed in the EPR spectra, demonstrating the efficient production of ^1^O_2_, •OH and O_2_^•−^ by BTIC‐*δ*OH‐2Cl sensitizing.^[^ ^5^, ^41^ ^]^ The intracellular ROS levels introduced by the PDT treatment were examined by particular cell‐permeable fluorogenic probes (Figure 3g). 2′,7′‐dichlorodihydrofluorescein diacetate (DCFH‐DA) is a fluorescence probe for the determination of the degree of overall oxidative stress. SOSG, APF, and dihydroethidium (DHE) were selective probes for ^1^O_2_, •OH, and O_2_^•−^, respectively. Those probes are non‐fluorescent but can emit strong green or red fluorescence when reacting with corresponding ROS species. The confocal laser scanning microscope (CLSM) results demonstrated the PDT treatment via laser irradiation of BTIC‐*δ*OH‐2Cl NPs could perform well in cells, leading to significantly increased intracellular levels of ^1^O_2_, •OH, and O_2_^•−^.

**Figure 5:**
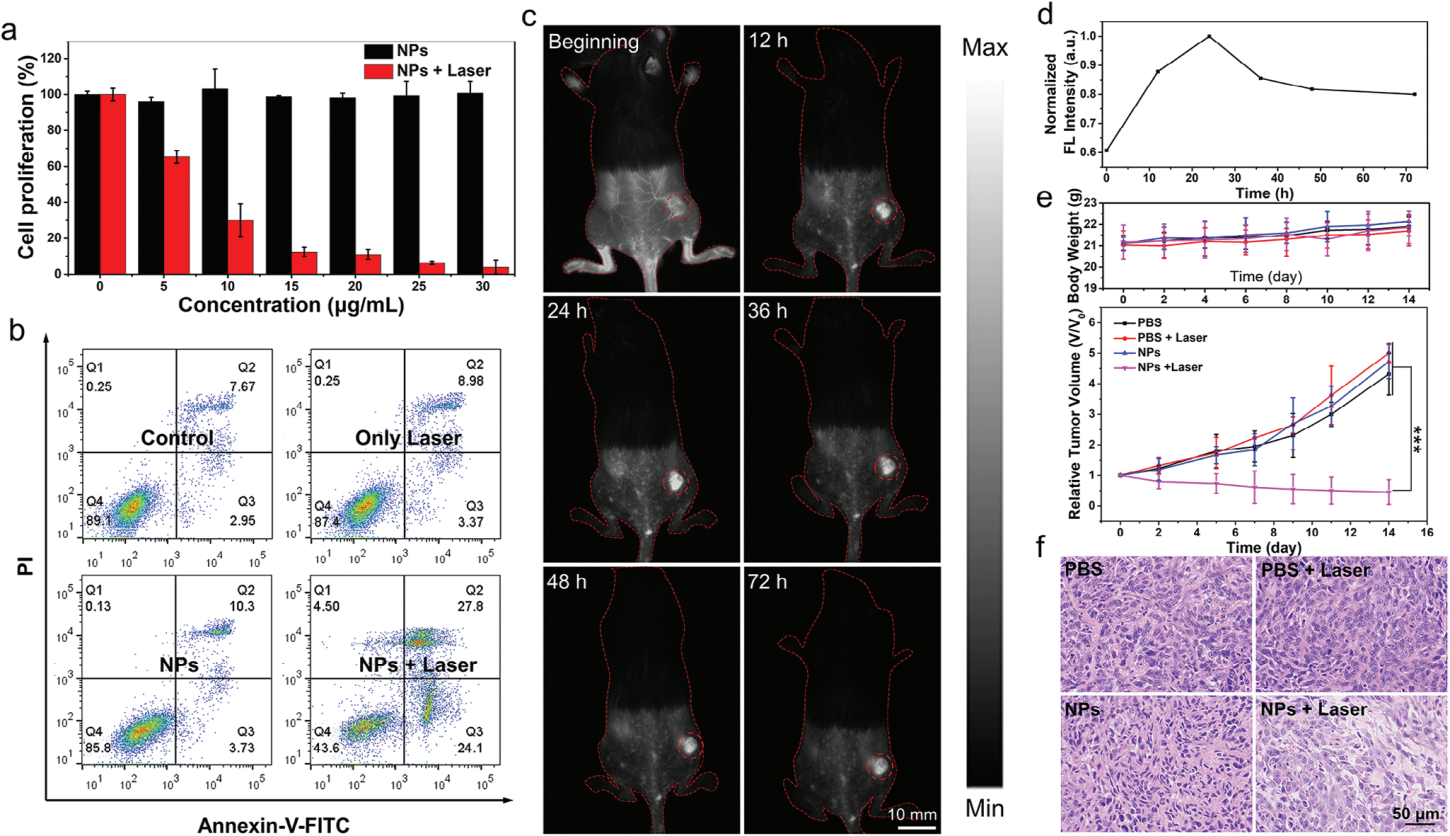
a) Cell proliferation of 4T1 cells incubated with BTIC‐*δ*OH‐2Cl NPs at various concentrations in the dark and after 360 J cm^−2^ NIR light irradiation (808 nm, 0.6 W cm^−2^ for 10 min). b) Apoptosis and necrosis analysis using flow cytometry toward 4T1 cells after different treatments. NIR light irradiation (808 nm, 0.6 W cm^−2^, 10 min) was conducted after cells were incubated with BTIC‐*δ*OH‐2Cl NPs (10 µg mL^−1^). c) In vivo NIR‐II FLI of tumors after 4T1 tumor‐bearing mice were intravenously injected with BTIC‐*δ*OH‐2Cl NPs at different time intervals. The outline of the mice is indicated by a red dashed line, and the tumor location is marked with a red dashed circle. d) Semi‐quantified NIR‐II fluorescence intensities of tumor region at different times post‐administration from (c). e) Body weight changes and tumor growth profile of 4T1 tumor‐bearing mice with different treatments. f) H&E staining slices of tumors collected from 4T1 tumor‐bearing mice 24 h after receiving the various treatments. Statistical significance: ^*^ *p* < 0.05, ^**^ *p* < 0.01, and ^***^ *p* < 0.001. Error bars are the standard deviation of the mean (*n* = 3).

Further, the uptake behavior of the NPs by 4T1 tumor cells was studied to inspect dark‐ and photo‐toxicity (Figure S24, Supporting Information). After that, the anti‐tumor efficiency of PDT was explored by CCK‐8 and live/dead cell co‐staining assays. As shown in **Figure** **5**a, BTIC‐OH‐2Cl NPs were found to have no obvious cytotoxicity to 4T1 cells up to 30 µg mL^−1^ under dark conditions. Upon 360 J cm^−2^ laser irradiation (808 nm, 0.6 W cm^−2^ for 10 min), the plot of cell viability versus concentration manifested that BTIC‐*δ*OH‐2Cl NPs showed an efficient tumoricidal effect to 4T1 cells with an IC_50_ value (the concentration causing 50% growth inhibition) of 7.7 µg mL^−1^. The photo‐cytotoxicity should come from the combined impact of •OH and ^1^O_2_ produced by BTIC‐*δ*OH‐2Cl NPs upon laser irradiation. After that, pronounced phototoxicity toward the cancer cells in hypoxic conditions was revealed (Figure S25, Supporting Information), demonstrating the validity of BTIC‐δOH‐2Cl NPs in the tumor‐hypoxic environments. Cytotoxicity was also analyzed by calcein acetoxymethyl ester (Calcein‐AM)/propidium iodide (PI) assay to differently stain living cells (green) from dead cells (red). As shown in Figure S26 (Supporting Information), the control and dark‐cytotoxicity groups of BTIC‐*δ*OH‐2Cl NPs showed strong green fluorescence (living cells) but no red fluorescence (dead cells). In contrast, the laser‐irradiation group of BTIC‐*δ*OH‐2Cl NPs showed an overwhelming majority of dead cells. Moreover, apoptosis analysis on flow cytometry (Figure 5b) revealed that the PDT treatment would induce both early apoptosis (Q3 zone) and late apoptosis (Q2 zone) of 4T1 cells.

Encouraged by its excellent performance in cell experiments, we continued to evaluate the PDT efficacy of BTIC‐*δ*OH‐2Cl NPs in vivo. Semi‐quantitative analysis showed that BTIC‐*δ*OH‐2Cl NPs exhibited a sufficient blood circulation half‐life of 3.0 h (Figure S27, Supporting Information). Accordingly, obvious fluorescence could be observed at the tumor site 12 h‐post tail‐vein injection of BTIC‐*δ*OH‐2Cl NPs (0.5 mg mL^−1^, 0.2 mL), which further increased and reached the maximum at 24 h (Figure 5c,d). NIR‐II imaging is an effective method for observing the progress of tumor growth. We observed the trend of tumor growth through NIR‐II imaging of tumor‐bearing mice injected via the tail vein (Figure S28, Supporting Information). The long blood circulation time and tumor retention properties of BTIC‐*δ*OH‐2Cl NPs will benefit the PDT applications (Figure 5e). We next evaluated the PDT performance of BTIC‐*δ*OH‐2Cl NPs on the 4T1 tumor‐bearing mice over a 14‐day follow‐up period. At the optimized enrichment time (24 h‐post injection), 540 J cm^−2^ laser irradiation (808 nm, 0.6 W cm^−2^ for 15 min) was performed at the tumor site, which was designated as the NPs + laser group. The results demonstrated the treatment could significantly inhibit tumor growth. On the contrary, for the mice treated with NPs alone (without irradiation) and PBS controls (with and without irradiation), the tumors in all these three groups gradually increased by 4 times after 14 days (Figure 5e; Figure S29, Supporting Information). We also monitored the photothermal effect during the laser irradiation process, and only a slight temperature elevation (≈5 °C) was observed (Figure S30, Supporting Information), Moreover, the photothermal conversion efficiency was determined to be 55%, 48%, and 52% for BTIC‐4Cl, BTIC‐OH‐*δ*, BTIC‐*δ*OH‐2Cl NPs, as showed in Figure S31 (Supporting Information). It confirmed that the antitumor effect was mainly caused by the photochemical reactions of PDT treatment. To further validate the outcome of antitumor treatment, the tumors were sectioned and proceeded to the histological analysis by hematoxylin and eosin (H&E) stain (Figure 5f). The results showed that the tumor cells in the control group were closely arranged and had no obvious damage, while the tumor cells in the NPs + laser group were sparsely arranged with a large number of necrotic tumor cells, which confirmed the effective destruction of tumor cells by the PDT treatment. Moreover, no significant weight loss was observed during the 14‐day treatment (Figure 5e). The in vivo biodistribution of BTIC‐*δ*OH‐2Cl NPs in the main organs of 4T1 tumor‐bearing mice 24 h‐post injection was determined by NIR‐II fluorescence (Figure S32, Supporting Information). Histological H&E staining analysis of major organs collected from the mice received the 14‐day treatments. No significant damage to the main organs, including the heart, liver, spleen, lung, and kidney, was observed (Figure S33, Supporting Information). In addition, after PDT treatment, the white blood cells and central mitochondrial cells of the cured mice were found to decrease (Table S1, Supporting Information). All these results demonstrated that BTIC‐*δ*OH‐2Cl NPs were safe and biocompatibility, with potential for NIR‐II FLI imaging‐guided PDT applications.

## 3 Conclusion

In conclusion, three π‐conjugated molecules, BTIC‐4Cl, BTIC‐OH‐*δ*, and BTIC‐*δ*OH‐2Cl with different end groups, were synthesized, and the end‐group effects on their π–π electron coupling in the aggregated state and phototheranostic properties were investigated. The optical spectra and single crystal X‐ray diffraction analysis revealed that the hydrogen‐bonding interactions in BTIC‐OH‐*δ* induced a more intense π–π electron coupling than the chlorine‐mediated interactions in BTIC‐4Cl. BTIC‐OH‐*δ* NPs that favored H‐aggregation showed a more intense π–π coupling, which lowered fluorescence efficiency but benefited PDT efficiency. Therefore, they showed fluorescence quantum yields of 3.76% (BTIC‐4Cl), 1.95% (BTIC‐OH‐*δ*), and 2.27% (BTIC‐*δ*OH‐2Cl), respectively; reversely, their ROS yields were determined to be 3.3 (BTIC‐4Cl), 25.9 (BTIC‐OH‐*δ*), and 14.6 (BTIC‐*δ*OH‐2Cl) folds of ICG. The reason for PDT enhancement was assumed that the stronger π–π electron coupling would facilitate energy and electron transfer to the surface of NPs to promote the photochemical reactions between the encapsulated PSs and O_2_ or H_2_O in the environment. Further, BTIC‐*δ*OH‐2Cl NPs that showed balanced fluorescence and PDT properties were selected for cellular and in vivo characterizations. NIR‐II fluorescence imaging with a high SBR of 1.63 and a notable PDT‐based tumor suppression effect was realized using BTIC‐*δ*OH‐2Cl NPs as the photosensitizing agent. Overall, the results demonstrate that this end‐group strategy is viable for rational tuning the properties of large and highly hydrophobic π‐conjugated molecules for improved phototheranostic applications.

## Conflict of Interest

The authors declare no conflict of interest.

## Supporting information

Supporting Information

Supporting Information

## Data Availability

The data that support the findings of this study are available from the corresponding author upon reasonable request.
