# Supplementary material for: The Balance Effect of π–π Electronic Coupling on NIR‐II Emission and Photodynamic Properties of Highly Hydrophobic Conjugated Photosensitizers
Source: Adv Sci (Weinh). 2023 Dec 28;11(6):2307569. doi: 10.1002/advs.202307569 (PMC10853711; doi:10.1002/advs.202307569)
Supplement: Supplementary file 1 — Supporting Information [file ADVS-11-2307569-s001.pdf]

ADVANCED SCIENCE
Open Access

Supporting Information

for *Adv. Sci.*, DOI 10.1002/advs.202307569

The Balance Effect of π–π Electronic Coupling on NIR-II Emission and Photodynamic Properties of Highly Hydrophobic Conjugated Photosensitizers

Yulin Zhu, Hanjian Lai, Ying Gu, Zixiang Wei, Lin Chen, Xue Lai, Liang Han, Pu Tan, Mingrui Pu, Fan Xiao, Feng He and Leilei Tian**

Supporting Information for

The Balance Effect of π-π Electronic Coupling on NIR-II Emission and Photodynamic Properties of Highly Hydrophobic Conjugated Photosensitizers

Yulin Zhu, Hanjian Lai, Ying Gu, Zixiang Wei, Lin Chen, Xue Lai, Liang Han, Pu Tan, Mingrui Pu, Fan Xiao, Feng He and Leilei Tian**

Y. Zhu, H. Lai, Y. Gu, L. Chen, X. Lai, Dr. L. Han, P. Tan, M. Pu, Prof. F. He
Shenzhen Grubbs Institute and Department of Chemistry, Southern University of Science and Technology, Shenzhen 518055, China.
E-mail: hef@sustech.edu.cn

Y. Zhu, Y. Gu, Z. Wei, L. Chen, Dr. L. Han, F. Xiao, Prof. L. Tian
Department of Materials Science and Engineering, Southern University of Science and Technology, Shenzhen 518055, China.
E-mail: tianll@sustech.edu.cn

Y. Zhu, X. Lai

Z. School of Chemistry and Chemical Engineering, Harbin Institute of Technology, Harbin 150001, China.

1. General Methods

Instruments. ^1H NMR spectra were recorded on a Bruker Advance 400 instrument in $CDCl_3$ or CD_3ClCD_3Cl using tetramethylsilane as internal standard. TEM images were carried out on Hitachi HT7700 (Hitachi, Japan). Cyclic voltammetry (CV) measurements were performed on a CHI 660E potentiostat/galvanostat (Shanghai Chenhua Instrumental Co., Ltd. China) to determine the HOMO and LUMO levels of the molecules, in an acetonitrile solution of 0.1 mol

L^{-1} tetrabutylammonium hexafluorophosphate ($[n\text{-}Bu4N]^+[PF6]^-$) at a potential scan rate of 100 mV s^{-1} with an Ag/Ag^+ reference electrode and a platinum wire counter electrode under a argon atmosphere. The XPS measurement was tested by ULVAC PHI 5000 Versa Probe III with Al Kα radiation (1486.6 eV). Hydrodynamic diameters and zeta potentials of the nanoparticles were determined by a Nano Brook Zeta PALS potential analyzer (Brookhaven Instruments Corporation, USA) in ultrapure water. Electron paramagnetic resonance (EPR) spectra were collected with a Bruker EMXplus EMXmicro EPR. The 808 nm continuous-wave laser systems were purchased from Changchun Laser Technology Co., Ltd. (Changchun, China). The output power of the lasers was measured with a VLP-2000 laser power meter (Xi'an Hirsh Laser Tech. Co., Ltd., China). CCK8 assay result and the DPBF absorption decay in the reactive oxygen species (ROS) detection and the fluorescence intensity changes of APF and SOSG in the ROS detection were recorded by a Cytation 3 microplate reader (BioTek Instruments, Inc., Winooski, USA). The fluorescence images of cells in the intracellular ROS detection and the fluorescence images of live cells and dead cells were taken by a Leica TCS-SP8 laser scanning confocal microscope (LSCM, Leica, Germany). UV-Vis-NIR absorption spectra were recorded on a UV3600 spectrometer (Shimadzu, Japan), and their fluorescence spectra were taken by an iHR320 imaging spectrometer (Horiba, Japan). The H&E staining of slices were examined by an Eclipse ci optical microscope (Nikon, Japan). Apoptosis and necrosis analysis were recorded by flow cytometry (FACSCanto, Becton, Dickinson and Company, USA). In vivo NIR-II fluorescence imaging was collected with NIR-OPTICS Series III 900/1700 small animal imaging system (Suzhou NIR-Optics Technology Co., Ltd., China) equipped with a1000, 1100, 1250 or 1319 nm long-pass filter (1000/1100/1250/1319 LP) and 808 nm laser source. The IR thermographic images were taken by a near infrared thermal imager (FLIR-E64501, FLIR Systems Inc., USA). The blood routine test were conducted on the automatic hematology analyzer (DF52Vet, Dymind Biotech., China).

Chemicals and materials. Chemicals and solvents were utilized as obtained with no additional

treatment unless specified otherwise. IC-2Cl purchace from Derthon Optoelectronic Materials Science Technology Co., Ltd. 1,2-distearoyl-sn-glycero-3-phosphoethanolamine-N-[methoxy(polyethylene glycol)-2000] (DSPE-PEG_{2000}) was purchased from Ponsure Biotech, Inc. (Shanghai, China). Phosphate buffered saline (PBS) was prepared by dissolving tablets obtained from Amresco (Solon, OH, USA) in ultrapure water according to the manufacturer's instructions (10.0 mM PBS, containing 137.0 mM Na^+ and 2.0 mM K^+). Chlorin e6 (Ce6) was purchased from Frontier Scientific, Inc. (USA). Cell Counting Kit-8 (CCK8) and indocyanine green (ICG) were purchased from Ponsure Biotechnology. (Shanghai, China) 2,7-Dichlorodihydrofluorescein diacetate (DCFH-DA), aminophenyl fluorescein (APF), and singlet oxygen sensor green (SOSG) were obtained from Shanghai Maokang Biotechnology Co., Ltd. (Shanghai, China). Dihydrorhodamine 123 (DHR 123) was purchased from Guangzhou Jietewei Biotechnology Co., Ltd (China). Dihydroethidium (DHE) was purchased from Guangzhou Zuoke Biotechnology Development Co., Ltd (China). 1,3-Diphenylisobenzofuran (DPBF) and 5,5-dimethyl-1-pyrroline N-oxide (DMPO) were purchased from Adamas Reagent Co., Ltd. (Shanghai, China). 2,2,6,6-Tetramethylpiperidine (TEMP) was purchased from TCI Chemical Industry Co., Ltd. (Japan). 2-(tert-butoxycarbonyl)-2-methyl-3,4-dihydro-2H-pyrrole 1-oxide (BMPO) were purchased from DOJINDO Chemical Technology Co., Ltd (Shanghai, China). Annexin V-FITC Apoptosis Detection Kit was purchased from Beyotime Biotech Co., Ltd. (Shanghai, China). Copper grid for transmission electron microscopy (TEM) detection was purchased from Zhongjingkeyi Technology Co., Ltd. (Beijing, China). Ultrapure water (at 18.2 MOhm) was produced by a Millipore synergy UV Ultrapure water purification system (MA, USA). 4T1 cells were purchased from Jennio Biotech Co., Ltd. (Guangzhou, China). Dulbecco's modified Eagle's media (DMEM), trypsin-EDTA solution, fetal bovine serum (FBS), and penicillin/streptomycin were purchased from Gibco BRL Co., Ltd. (Grand Island, NY, USA). Female BALB/c mice were purchased from Vital River Laboratory Animal Technology Co., Ltd. (Zhejiang, China).

Single crystal. Diffraction quality crystals were grown by solvent diffusion method. Acceptors (5 mg) was dissolved in CH_2Br_2 (5 mL) in the 15 mL sample bottle, then 10 mL of ethanol was added it slowly. The bottle was then sealed tightly, and left undisturbed for 10 days. A suitable crystal was selected and the crystal was mounted on a MITIGEN holder with oil on a 'Bruker D8 Venture' diffractometer. The crystal was kept at 100 K during data collection. The CCDC number of BTIC-4Cl is 2232974. The CCDC number of BTIC-OH-δ is 2232973.

Density functional theory calculations. To reduce the computational cost, ethylhexane groups were replaced by methyl groups. The ground-state (S_0) geometries of structure-simplified BTIC-OH-δ, BTIC-δOH-2Cl, and BTIC-4Cl were optimized at the B3LYP/6-31G(d) level[2].

Fabrication of NPs. In a typical process, a Delta ultrasonic cleaner (Model DC300H) operating at 100% amplitude was used to subject a mixture of 0.5 mg BTIC-4Cl and 5 mg DSPE-PEG2000 (10 times the weight of BTIC-4Cl) in 2.0 mL of tetrahydrofuran (THF) to ultrasonication for 1 minutes. The resulting solution was then added to 20.0 mL of ultrapure water under continued ultrasonication. Subsequently, THF was volatilized with a magnetic stirrer at 400 rpm at room temperature, and the product was further concentrated by ultrafiltration using an Amicon Ultra centrifugal filter unit with a molecular weight cutoff of 10 kDa and stored at 4 °C for further use. BTIC-OH-δ, BTIC-δOH-2Cl, and Ce6 NPs were prepared according to the same procedure.

Fluorescence quantum yield (PLQY) calculation. In order to measure the quantum yield of BTIC-OH-δ, BTIC-δOH-2Cl, and BTIC-4Cl NPs, a reference dye IR-26 was chosen. Five difference concentrations were measured and the integrated fluorescence (850-1500 nm region) was plotted against absorbance at 808 nm (the excitation wavelength) for both IR-26 and the samples. Comparison of the slopes led to the determination of the quantum yield of NPs. The quantum yield was calculated in the following manner:

$$\mathrm{PLQY_{sample}} = \mathrm{PLQY_{ref}} \frac{\mathrm{slope_{sample}}}{\mathrm{slope_{ref}}} \left(\frac{\mathrm{n_{sample}}}{\mathrm{n_{ref}}}\right)^2 \quad (1)$$

Where $PLQY_{ref}$ is 0.5%[3] and n_{sample} and n_{ref} are the refractive index of water and dichloroethane (DCE), respectively.

Detection of $^1O_2$, •OH, and $O_2^{•-}$ in EPR. TEMP, DMPO, and BMPO were used to detect $^1O_2$, •OH, and $O_2^{•-}$, respectively. To detect $^1O_2$ and •OH, 90 μL of BTIC-*δ*OH-2Cl NPs (100 μg mL^{-1}) were mixed with 10 μL of 1M TEMP/DMPO and irradiated with an 808 nm laser (1.0 W cm^{-2}, 5 min). For $O_2^{•-}$ detection, 100 μg mL^{-1} of BTIC-*δ*OH-2Cl NPs were dispersed in 100 μL water containing 25 mM of BMPO and 10% DMSO, and then irradiated with an 808 nm laser (1.0 W cm^{-2}, 5 min). TEMP, DMPO, and BMPO treated with the same laser dose were tested as controls.

ROS generation measurement. All experiments were carried out in 96 well plates. The total ROS, $^1O_2$, •OH, and $O_2^{•-}$ were probed by DPBF, SOSG, APF, and, DHR123. The test solution with optical density equal to 0.1 for DPBF experiment and 0.2 for the SOSG, APF, and DHR123 experiment. In the total ROS experiment, the DPBF concentration was 100 μg mL^{-1}. The absorption signal was measured between 350-500 nm after 0.6 W cm^{-2} 808nm laser irradiation. In the $^1O_2$ experiment, the SOSG concentration was 5 μM. Fluorescence was measured between 510-700 nm using an excitation wavelength of 480 nm after 0.6 W cm^{-2} 808nm laser irradiation. In the •OH experiment, the APF concentration was 5 μM. Fluorescence was measured between 510-650 nm using an excitation wavelength of 480 nm after 0.6 W cm^{-2} 808nm laser irradiation. In the $O_2^{•-}$ experiment, the DHR123 concentration was 10 μM. Fluorescence was measured between 525-600 nm using an excitation wavelength of 480 nm after 0.6 W cm^{-2} 808nm laser irradiation.

ROS quantum yield (Φ_Δ) calculation. As for ROS quantum yield (Φ_Δ) calculation, Φ_Δ was calculated via the following equation:

$$\Phi_\Delta = \Phi_{\Delta\,ICG}\frac{S}{S_{ICG}} \qquad (2)$$

$\Phi_{\Delta\,ICG}$=0.2%[4], S represents slope of absorbance plot of DPBF vs irradiation time.

Electron Mobility Measurement. Mobility of electron was measured according to a field-independent space charge limited current (SCLC) model following the Mott-Gurney law: $J = \frac{9}{8}\varepsilon_0\varepsilon_r\mu\frac{V^2}{d^3}$, wherein the J is current, μ is the mobility, ε_0 is the permittivity of free space, ε_r is the relative permittivity of the material, d is the thickness and V is the voltage. The electron-only device structure is ITO/ZnO/active layer/PNDIT-F3N/Ag.

Cell culture. 4T1 cells were maintained as monolayer cultures in DMEM at 37 °C in an INCO2/153 CO_2 incubator (Memmert, Germany; 5% CO_2), the medias were supplemented with 10% FBS and 1% penicillin/streptomycin. The cells were cultured until confluence was reached before each experiment.

Dark toxicity and phototoxicity. The dark toxicity of BTIC-*δ*OH-2Cl NPs was evaluated as follows: After cell attachment, the culture media was replaced with 100 μL of fresh one containing different concentrations of BTIC-*δ*OH-2Cl NPs, followed by incubation for another 24 h. The cell viability was assessed by the widely used CCK8 test. The phototoxicity of BTIC-*δ*OH-2Cl NPs was evaluated according to a similar procedure, but the culture media was replaced with 100 μL of fresh one containing different concentrations of BTIC-*δ*OH-2Cl NPs, followed by exposed to 808 nm laser irradiation at 0.6 W cm^{-2} for 10 min. Irradiated cells were then incubated at 37 °C for another 24 h and cell viability was also evaluated using the CCK8 test. All data are based on three parallel experiments.

Intracellular ROS detection. 4T1 cells were incubated with 25 μg mL^{-1} of BTIC-*δ*OH-2Cl NPs (based on BTIC-*δ*OH-2Cl molecule) for 8 h followed by incubation with 10 μM DCFH-DA (total ROS), 2 μM SOSG ($^1O_2$), 10 μM APF (•OH), or 10 μM DHE ($O_2^{\bullet -}$) for 30 min. After being washed by PBS buffer, cells were irradiated with 808 nm laser at a power density of 0.6 W cm^{-2} for 10 min. Subsequently, the fluorescence was immediately observed using CLSM with the excitation wavelength of 488 nm, and emission collection wavelength was 510-550 nm for DCFH-DA, 500-550 nm for SOSG, 510-600 nm for APF, and 580-650 nm for DHE.

Live/dead cells staining assay. 4T1 cells were seeded in a confocal dish with a density of 1.3 × 10^4 cells per well. After 24 hours of cultivation, the medium was replaced by DMEM solution with 15 μg mL^{-1} BTIC-*δ*OH-2Cl NPs. After incubated for 8 h, the cells were applied to 808 nm laser irradiation (0.6 W cm^{-2}, 10 min). The confocal dish was placed in the incubator again for 2 h. After that, 4T1 cells were washed with PBS to wash away the NPs. Subsequently, the cells were staining with Calcein-AM and PI for 20 min in the incubator according to the product manual. Finally, the fluorescence was immediately observed using Leica confocal microscope.

Flow cytometry. After the cells were attached, the pure medium was replaced by the medium containing BTIC-*δ*OH-2Cl NPs (10 μg mL^{-1}). After 4 hours of culture, the cells were gently washed with PBS buffer for three times to remove the non-invasive samples; Next, the cells were irradiated with 808 nm laser (0.6 w cm^{-2}, 10 min), and the cells were further cultured for 6 h (37 °C, 5% CO_2). Finally, annexin V-FITC apoptosis detection kit and flow cytometry were used to detect apoptosis and necrosis.

Photothermal conversion efficiency of NPs. The NPs in water (1 ml with the absorbance at 808 nm were 0.2) were placed on a 48 well plate and exposed to 808 nm radiation of 0.8 w for 20 min to achieve a plateau. Subsequently, the laser was removed, and the solution was cooled down to room temperature. During this process, the solution temperatures were continuously recorded by an IR image camera. PCE was then calculated based on the reported method in the literature.[3]

$$\sum_i m_i C_{p,i} \frac{dT}{dt} = Q_{total} = Q_{sample} + Q_{dis} \qquad (1)$$

$$Q_{sample} = I(1 - 10^{-A_\lambda})\eta \qquad (2)$$

I: the laser power;

A_λ: the absorbance of the sample at the excitation wavelength;

η: the photo-thermal conversion efficiency;

Q_{sample}: the photo-thermal energy absorbed by the sample per second;

Q_{dis}: the heat dissipated from the laser mediated by the solvent and container;

$$Q_{total} = hA\Delta T_{max,mix} \quad (3)$$

$$Q_{dis} = hA\Delta T_{max,H_2O} \quad (4)$$

h: the heat transfer coefficient;

A: the surface area of the container;

$$\eta = \frac{hA(\Delta T_{max,mix} - \Delta T_{max,H_2O})}{I(1-10^{-A_\lambda})} \quad (5)$$

In this equation, only hA is unknown. In order to get the hA, we introduce θ.

$$\theta = \frac{\Delta T}{\Delta T_{max}} = \frac{T - T_{surrounding}}{T_{max} - T_{surrounding}} \quad (6)$$

$$\frac{d\theta}{dt} = \frac{hA}{\sum_i m_i C_{p,i}}\left[\frac{Q_{sample} + Q_{dis}}{hA\Delta T_{max}} - \theta\right] \quad (7)$$

When the laser was shut off, $Q_{sample} + Q_{dis} = 0$, equation (7) changes to:

$$\frac{d\theta}{dt} = -\frac{hA}{\sum_i m_i C_{p,i}} \ln\theta \quad (8)$$

Integration of equation (8)

$$t = -\frac{\sum_i m_i C_{p,i}}{hA} \ln\theta \quad (9)$$

Thus, hA can be determined by applying the linear time data from the cooling period vs – lnθ. Substituting hA value into Eq.5, the photothermal conversion efficiency (η) of the sample can be calculated.

Compared with solvent, mass of NPs was too little. Generally, the specific heat of water is much higher than other materials. Consequently, the m_i and $C_{p,i}$ of NPs were neglected. $C_{p,water} = 4.2\ J/g$

$$t = -\frac{m_{water} C_{p,water}}{hA} \ln\theta \quad (10)$$

Animal model. All animal experiments were in accord with Institutional Animal Use and Care Regulations, according to protocol No. SUSTC-JY202102063, approved by the Laboratory Animal Ethics Committee of the Southern University of Science and Technology. After being

acclimated and tested for infectious diseases for 1 week, 4-week-old BALB/c mice were subcutaneously injected with 4T1 cells (1×10^7 cells each mouse) at the flank region. After about one week, mice with tumor volumes of about 150-250 mm^3 were randomized into treatment groups. The tumor size was calculated using the following formula: Volume = (Length × $Width^2$)/2. 24 hours after the tail vein injection, 540 J cm^{-2} laser irradiation (808 nm, 0.6 W cm^{-2} for 15 min) was performed at the tumor site.

In vivo NIR-II fluorescence imaging. The tumor-bearing mouse was anesthetized with isoflurane to remove fur. Next, the aqueous solution (200 μL) of BTIC-δOH-2Cl NPs concentration equivalent to 0.5 mg·mL^{-1} was intravenously injected into the mouse. After injection, the mouse was imaged with the small animal imaging system at designated time points.

Histological analysis. For hematoxylin and eosin (H&E) staining, the tissues were fixed in 10% neutral buffered formalin, processed routinely into paraffin, sectioned at 4 μm, stained with H&E apoptosis assay kit and examined by microscope.

Blood routine test. The blood of the tumor-bear mice after 14-days PDT treatment were collected, and then blood routine test was conducted on the automatic hematology analyzer (DF52Vet, Dymind Biotech., China).

2. Experimental section

Synthesis of compound IC-mOH. To a solution of compound 4 (800 mg, 3.0 mmol, synthesized from previous work[1]) in 50 ml EtOH, 2 ml H_2O and 5 ml HCl were added, after reacting at room temperature for 1 h, the temperature was raised to 70 °C for 3 h. The reaction mixture was then cooled to room temperature and then extracted with CH_2Cl_2. The organic layer was washed with brine and dried over $MgSO_4$. The crude product was purified by flash column chromatography with CH_2Cl_2 as eluent to get the product IC-mOH (650 mg, 95.8 %). ^{1}H NMR

(400 MHz, $CDCl_3$) δ: 8.56 (d, *J*=8.6 Hz, 1H), 7.88-8.00 (m, 1H), 7.29-7.35 (m, 2H), 3.69-3.70 (m, 2H).

Synthesis of compound IC-OH-δ. The compound IC-mOH (600 mg, 2.7 mmol) was placed in 15 mL EtOH, and the temperature was raised to 70 ° C for complete dissolution. Then the solution was cooled to 4 °C and filtered to obtain the product IC-OH-*δ* (320 mg, 53.3 %). ^{1}H NMR (400 MHz, $CDCl_3$) δ: 8.00 (s, 1H), 7.89 (d, *J*=8.5 Hz, 1H), 7.31 (s, 1H), 3.69 (s, 2H).

Synthesis of compound BTIC-4Cl. A mixture of BT-2CHO (50 mg, 0.049 mmol), IC-2Cl (50 mg, 0.19 mmol), in $CHCl_3$/pyridine (20 ml/0.3 ml) was refluxed for 24 h under argon. The reaction mixture was then cooled to room temperature and extracted with $CHCl_3$. The organic layer was washed with brine and dried over $MgSO_4$. The crude product was purified by flash column chromatography with $CHCl_3$ as eluent to get the product BTIC-4Cl (60 mg, 80.8 %). ^{1}H NMR (400 MHz, $CDCl_3$) δ: 9.18 (s, 2H), 8.79 (s, 2H), 7.98 (s, 2H), 4.78 (d, *J*=8.0 Hz, 4H), 3.21-3.25 (m, 4H), 2.08-2.12 (m, 2H), 1.83-1.89 (m, 4H), 1.50-1.58 (m, 4H), 1.15-1.42 (m, 32H), 1.00-1.08 (m, 12H), 0.85-0.89 (m, 6H), 0.74-0.78 (m, 6H), 0.65-0.68 (m, 6H). MALDITOF-MS calcd for $C_{82}H_{86}Cl_4N_8O_2S_5$ (M^+): 1517.4235, found: 1516.4398.

Synthesis of compound BTIC-OH-δ. A mixture of BT-2CHO (50 mg, 0.049 mmol), IC-OH-*δ* (50 mg, 0.22 mmol), in THF/pyridine (20 ml/0.3 ml) was refluxed for 24 h under argon. The reaction mixture was then cooled to room temperature and extracted with $CHCl_3$. The organic layer was washed with brine and dried over $MgSO_4$. The crude product was purified by flash column chromatography with $CHCl_3$ as eluent to get the product BTIC-OH-*δ* (40 mg, 56.6 %). ^{1}H NMR (400 MHz, CD_3ClCD_3Cl) δ: 9.19 (s, 2H), 8.22 (s, 2H), 7.95 (d, *J*=8.2 Hz, 2H), 7.29 (d, *J*=10.2 Hz, 2H), 4.89 (d, *J*=7.7 Hz, 2H), 3.32-3.35 (m, 4H), 2.07-2.13 (m, 2H), 2.02-2.07 (m, 4H), 1.62-1.68 (m, 4H), 1.42-1.49 (m, 28H), 1.02-1.39 (m, 16H), 0.84-0.99 (m, 6H), 0.72-0.83 (m, 12H). MALDITOF-MS calcd for $C_{82}H_{90}N_8O_4S_5$ (M^+): 1410.5689, found: 1410.5410.

Synthesis of compound BTIC-δOH-2Cl. A mixture of BT-2CHO (200 mg, 0.20 mmol), IC-2Cl (50 mg, 0.19 mmol) in THF/pyridine (30 ml/0.5 ml) was reacted 6 hours under Argon at

50 °C, then the IC-OH-*δ* (42 mg, 0.19 mmol) was added for further 6 hours. The reaction mixture was then cooled to room temperature and then extracted with $CHCl_3$. The organic layer was washed with brine and dried over $MgSO_4$. The crude product was purified by flash column chromatography with chloroform as eluent and further purified by cycling preparative HPLC using a Japan Analytical Industry Co., ltd. LaboACE LC-7080 system equipped with a JAIGEL Silica based normal phase columns (10 μm, 20 mm x 250 mm) and a mobile phase consisting of $CHCl_3$. The flow rate was set at 3.5 mL/min to get the product BTIC-*δ*OH-2Cl (70 mg, 24.6 %). ^{1}H NMR (400 MHz, $CDCl_3$) δ: 9.19 (s, 1H), 9.12 (d, *J*=8.2 Hz, 1H), 8.80 (s, 1H), 8.64 (d, *J*=8.7 Hz, 1H), 8.14 (s, 1H), 7.97 (s, 1H), 7.87 (d, *J*=8.2 Hz, 1H), 7.35 (s, 1H), 4.76 (m, 4H), 3.23-3.24 (m, 4H), 2.08-2.19 (m, 2H), 1.88-1.99 (m, 4H), 0.65-1.54 (m, 66H). MALDITOF-MS calcd for $C_{82}H_{88}Cl_2N_8O_3S_5$ (M^+): 1462.4960, found: 1464.4609.

3. Supporting Figures and Tables

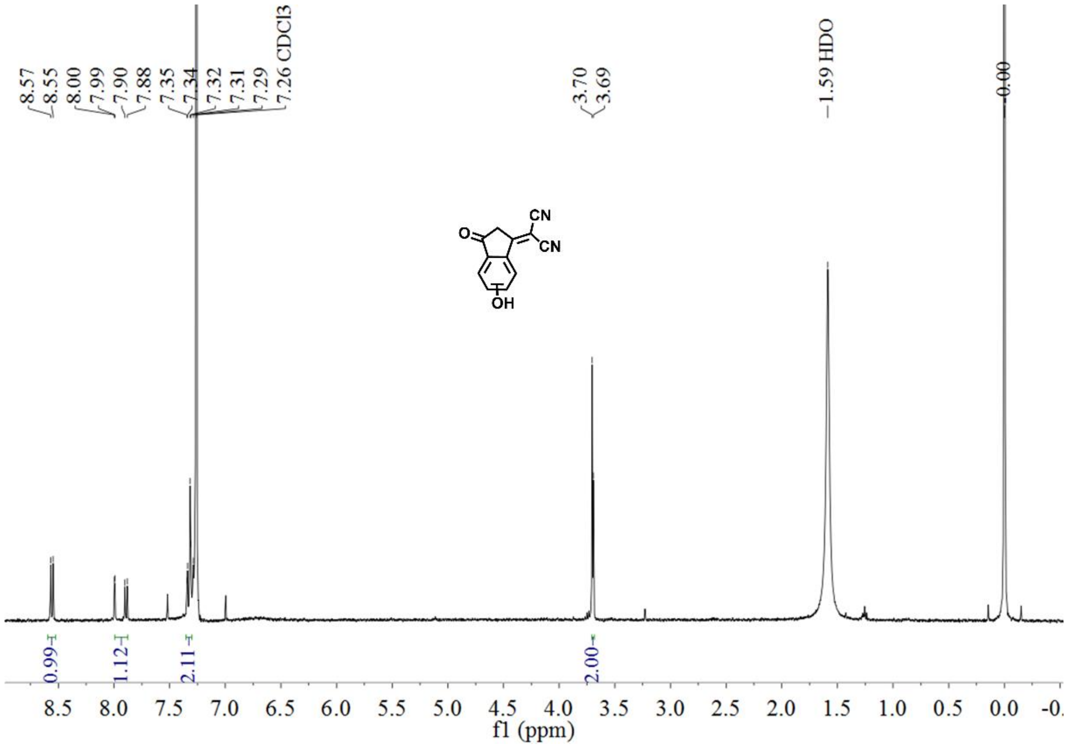

Figure S1. ^{1}H NMR spectrum of compound IC-mOH in $CDCl_3$.

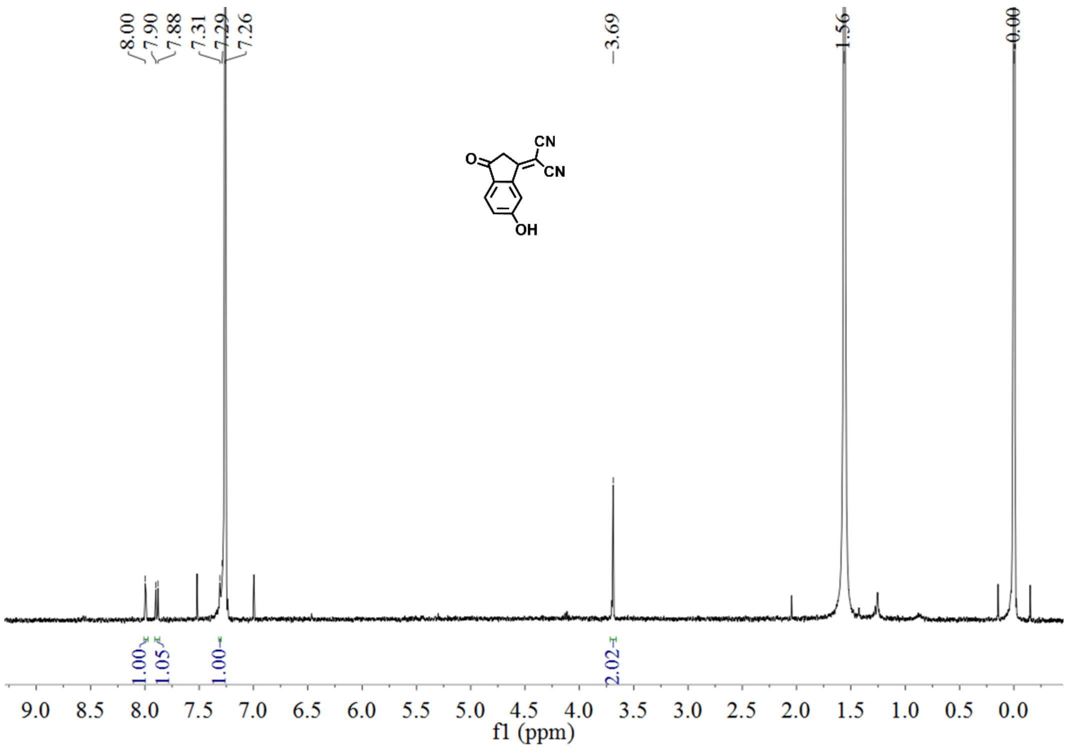

Figure S2. ^{1}H NMR spectrum of compound IC-OH-δ in $CDCl_3$.

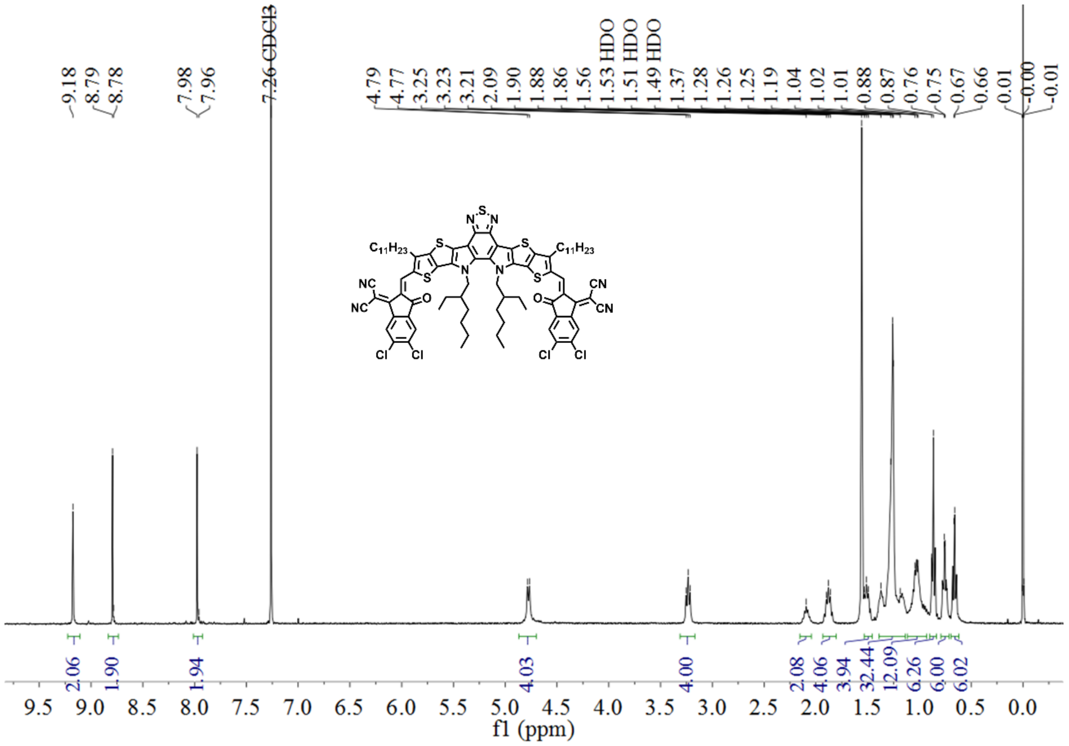

Figure S3. ^{1}H NMR spectrum of BTIC-4Cl in $CDCl_3$.

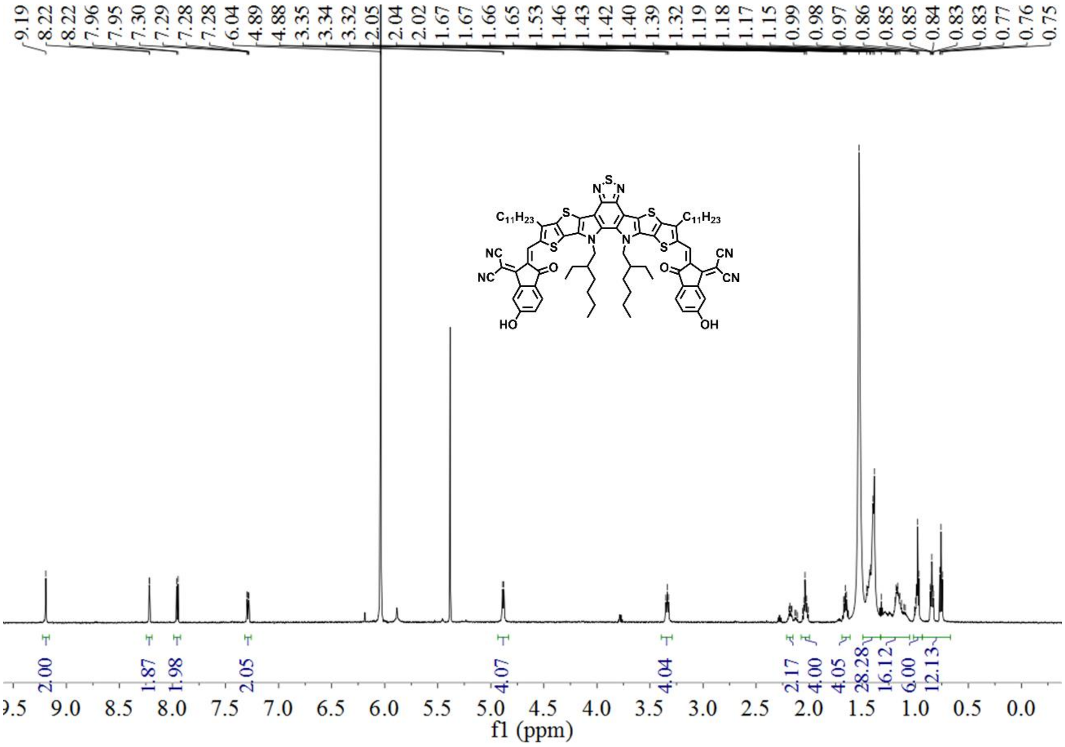

Figure S4. ^{1}H NMR spectrum of compound BTIC-OH-δ in CD_3ClCD_3Cl.

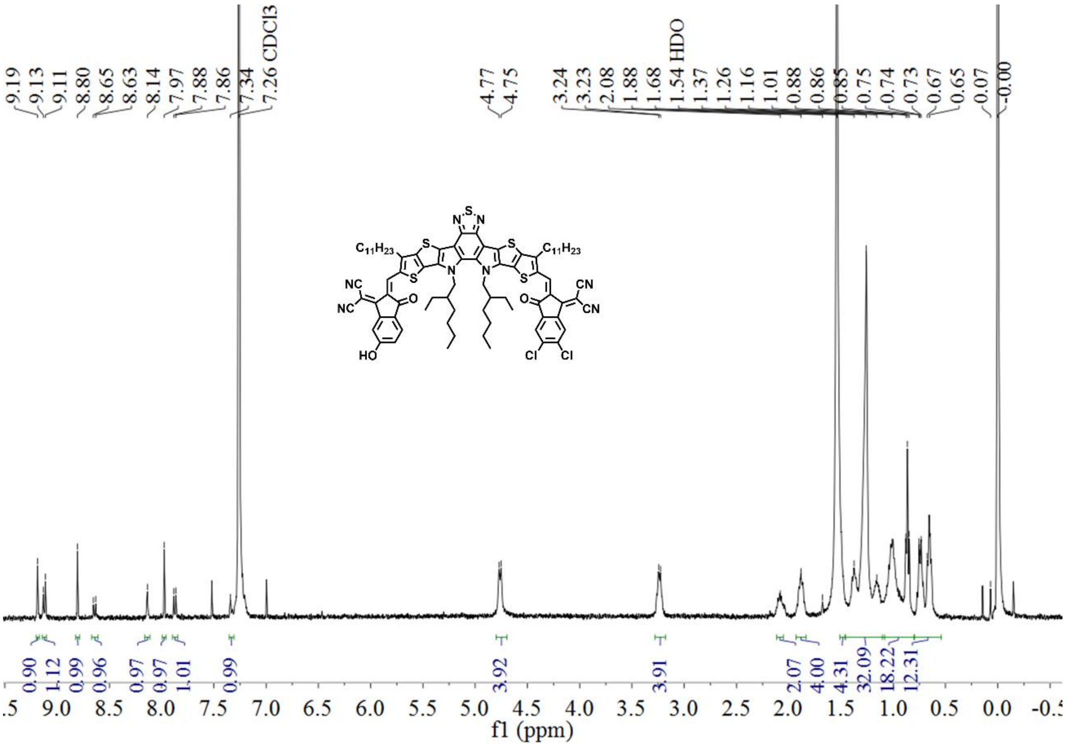

Figure S5. ^{1}H NMR spectrum of compound BTIC-δOH-2Cl in $CDCl_3$.

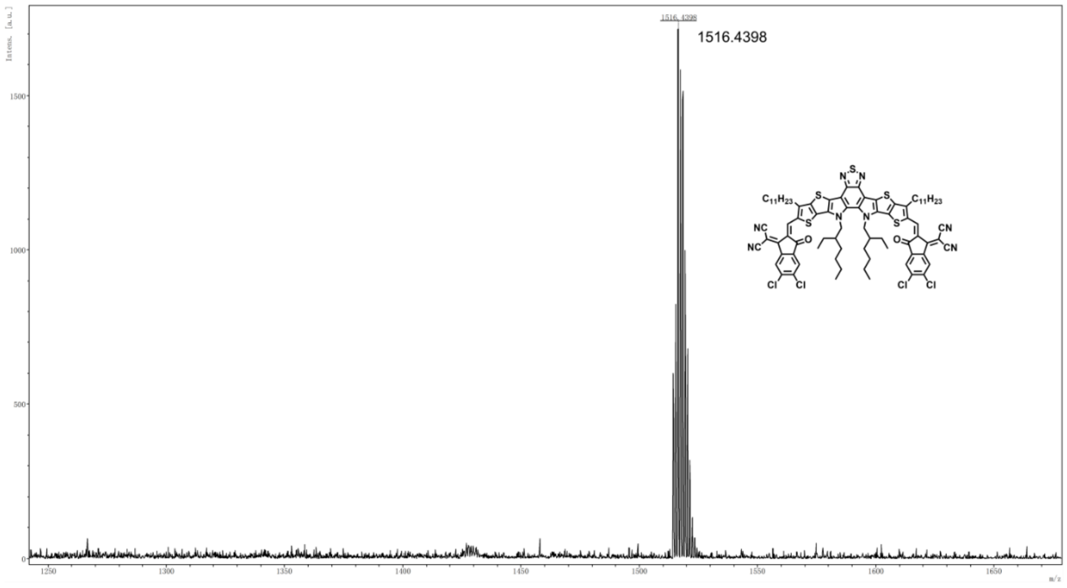

Figure S6. MALDI-TOF mass spectra of BTIC-4Cl.

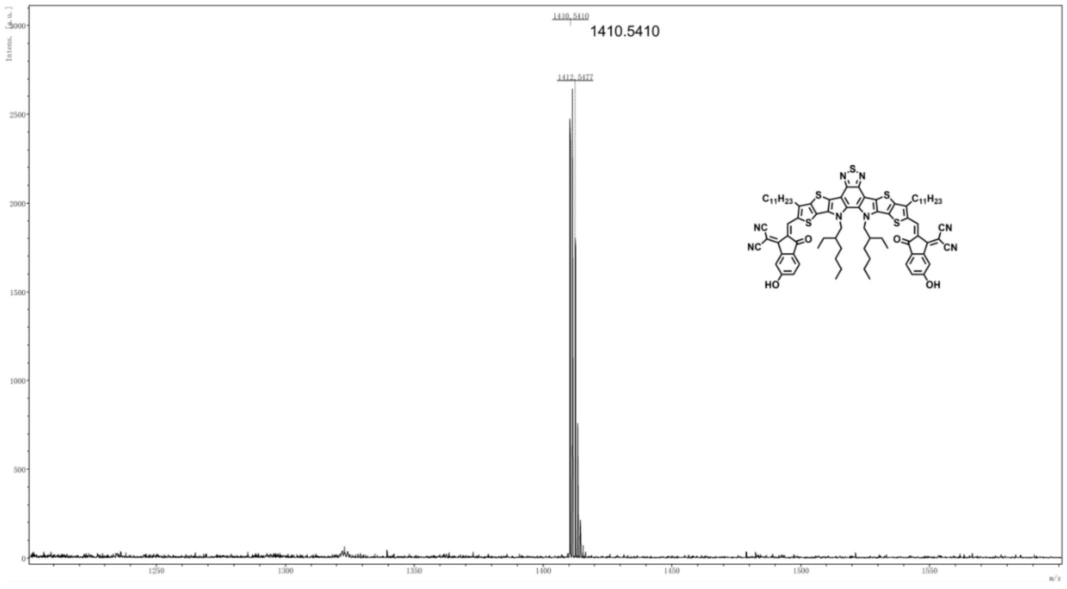

Figure S7. MALDI-TOF mass spectra of BTIC-OH-δ.

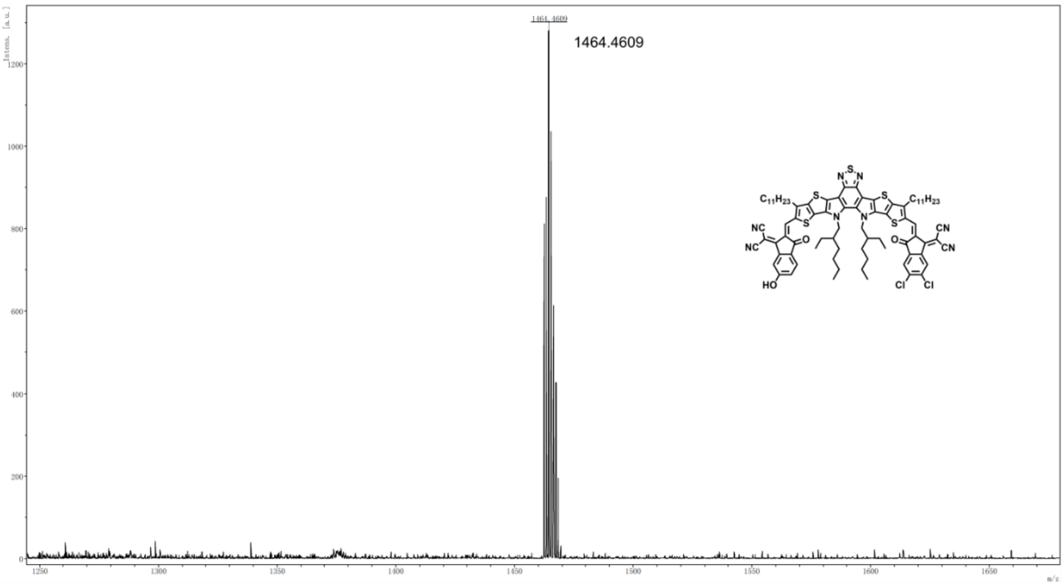

Figure S8. MALDI-TOF mass spectra of BTIC-δOH-2Cl.

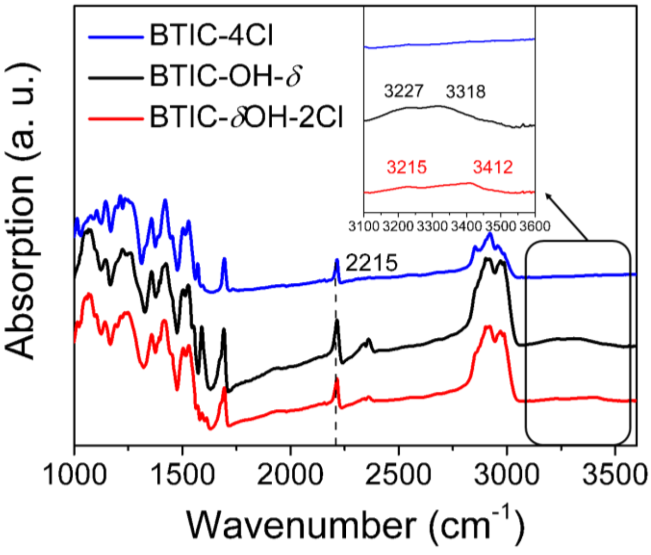

Figure S9. The infrared absorption spectra of BTIC-4Cl, BTIC-OH-δ, and BTIC-δOH-2Cl.

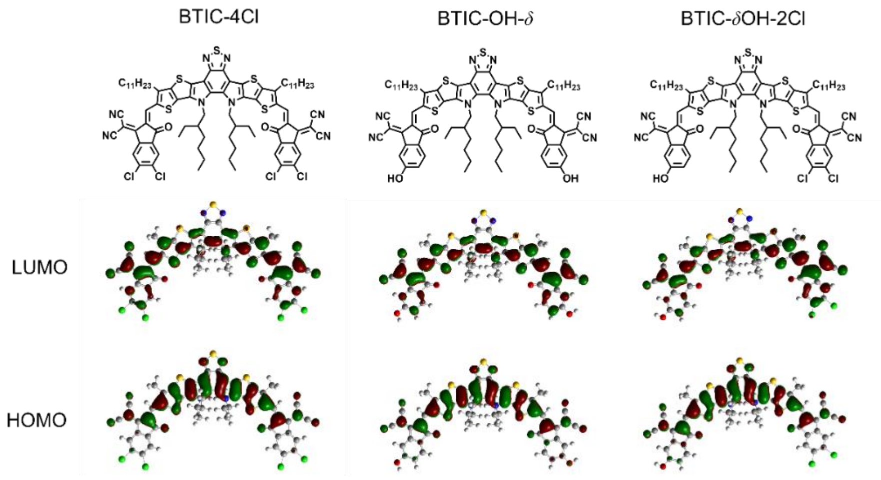

Figure S10. Calculated molecular orbital of BTIC-4Cl, BTIC-OH-δ, and BTIC-δOH-2Cl.

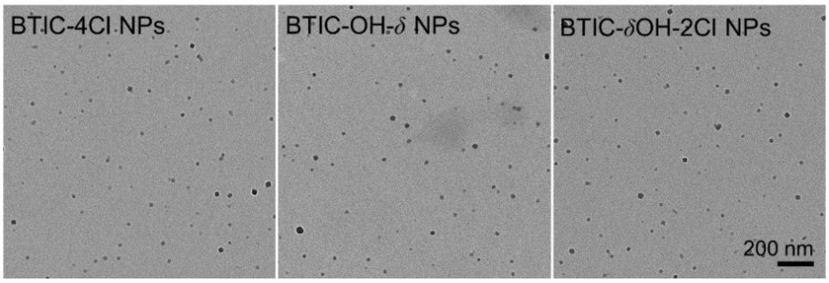

Figure S11. TEM image of BTIC-4Cl, BTIC-OH-δ, and BTIC-δOH-2Cl NPs in water.

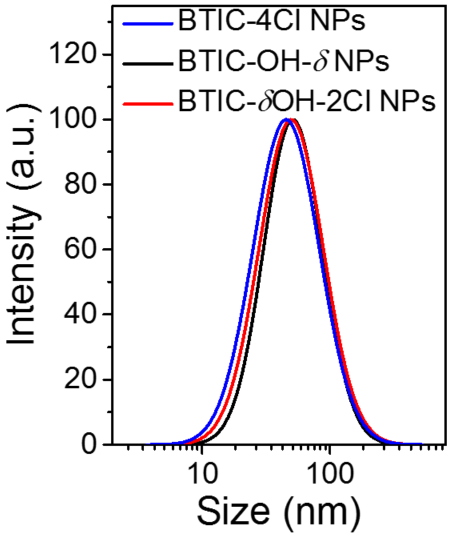

Figure S12. DLS analysis of BTIC-4Cl, BTIC-OH-*δ*, and BTIC-*δ*OH-2Cl NPs in ultra-pure water.

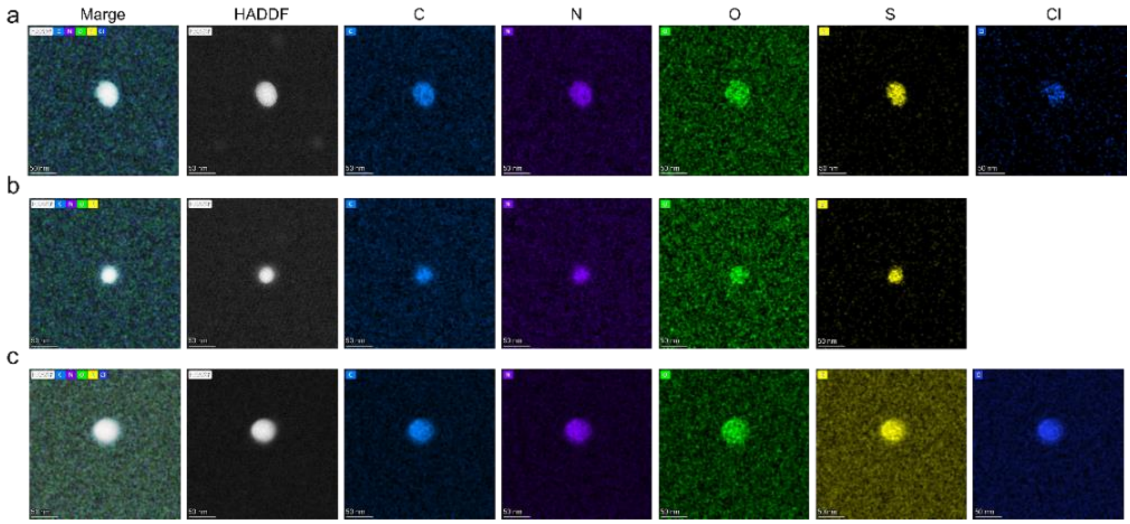

Figure S13. Elemental mappings of BTIC-4Cl (a), BTIC-OH-*δ* (b), and BTIC-*δ*OH-2Cl (c) NPs.

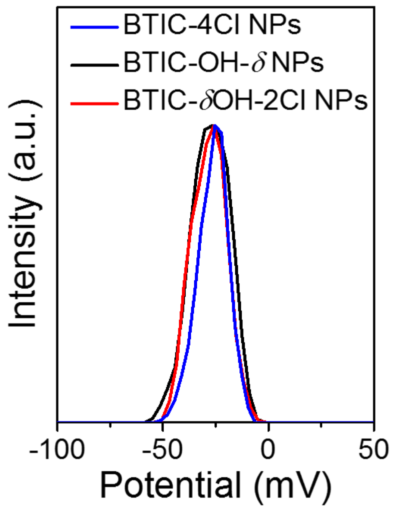

Figure S14. Zeta potentials of BTIC-4Cl, BTIC-OH-*δ*, and BTIC-*δ*OH-2Cl NPs in water.

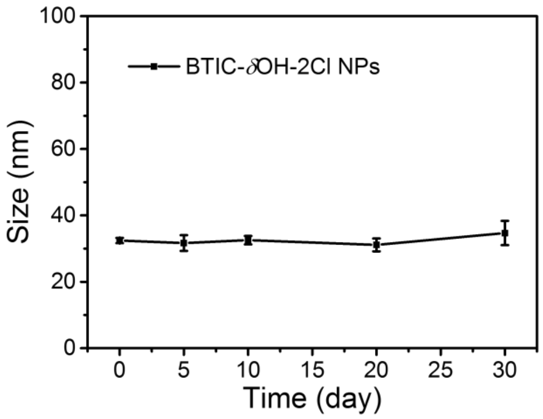

Figure S15. The DLS hydrodynamic diameters analysis results of BTIC-*δ*OH-2Cl NPs in PBS buffer for a period of 30 days. Error bars are the standard deviation of the mean (n=3).

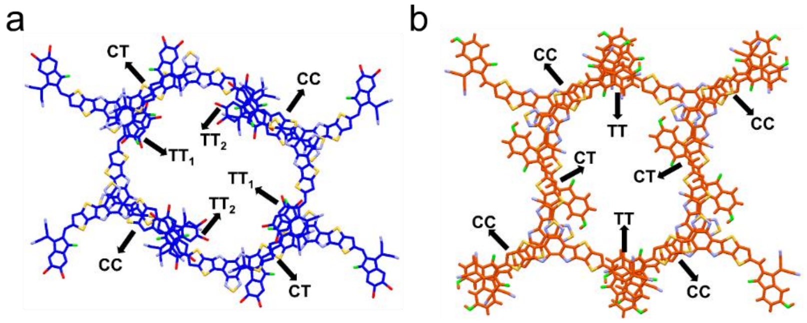

Figure S16. The single-crystalstructure of one elliptical frame of BTIC-4Cl (a) and BTIC-OH-*δ* (b).

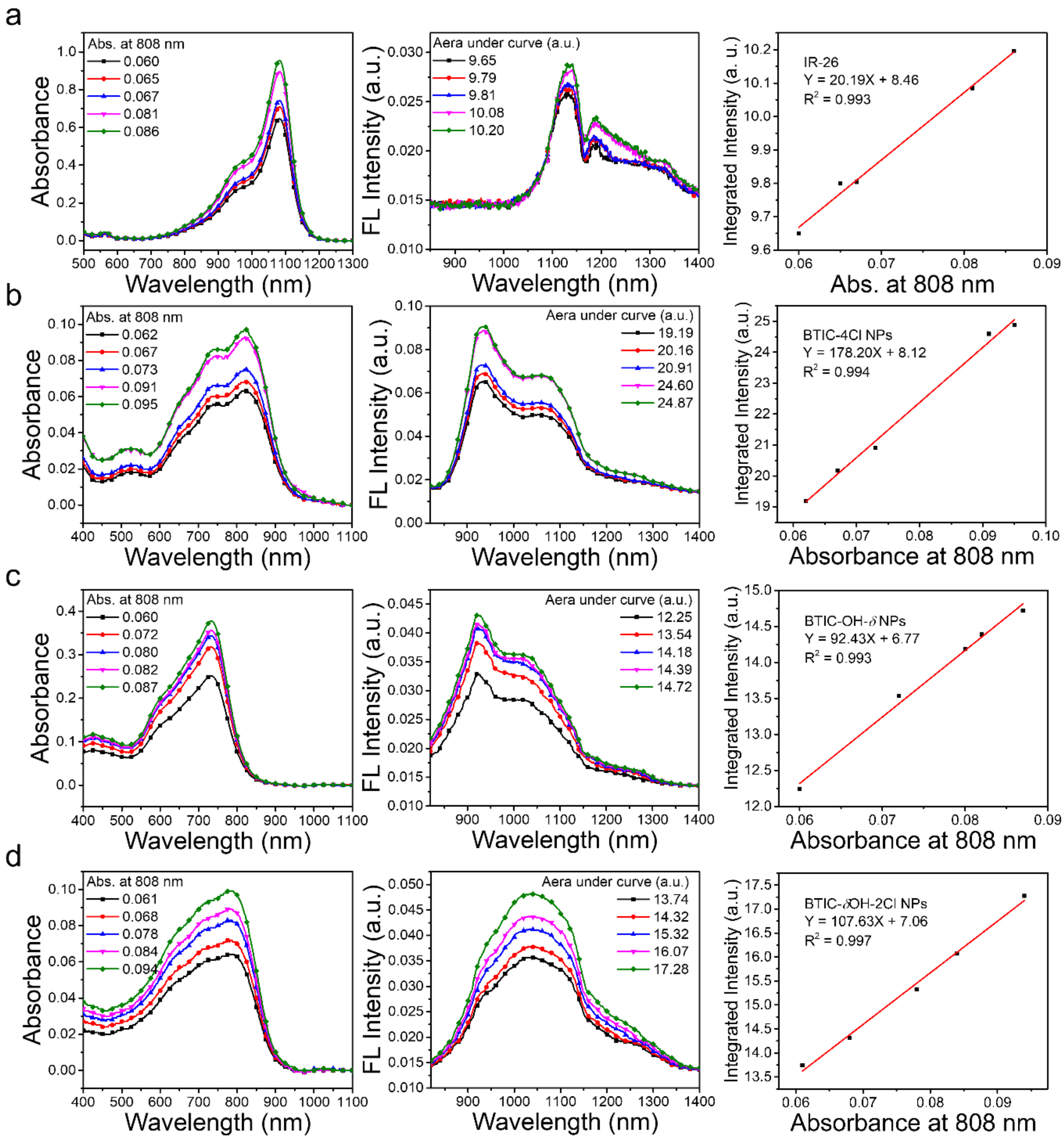

Figure S17. Data in NIR-II fluorescence quantum yield calculation. UV-Vis-NIR absorption spectra, PL spectra, and a plot of integrated NIR-II fluorescence intensity vs the absorbance at 808 nm of different samples: IR-26 in DCE (a), BTIC-4Cl NPs (b), BTIC-OH-*δ* NPs (c), and BTIC-*δ*OH-2Cl NPs (d) in ultrapure water. The fluorescence quantum yields of BTIC-4Cl, BTIC-OH-*δ*, and BTIC-*δ*OH-2Cl NPs were calculated to be 3.76 %, 1.95 %, and 2.27 % in ultrapure water.

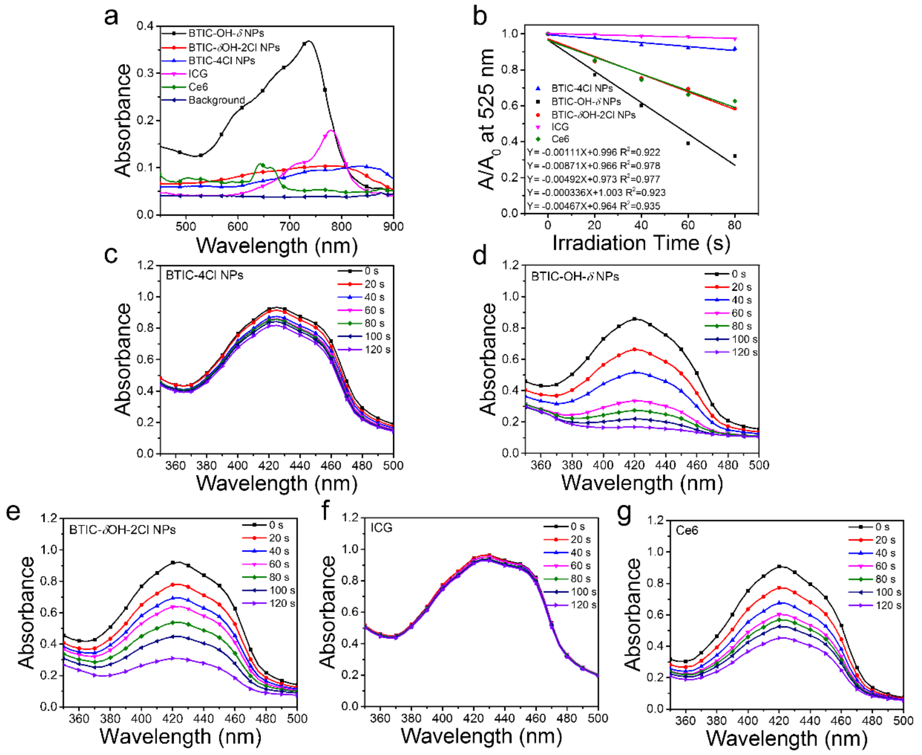

Figure S18. (a) UV-vis absorption of BTIC-4Cl NPs, BTIC-OH-*δ* NPs, BTIC-*δ*OH-2Cl NPs, ICG, Ce6, and background. (b) Linear plot of time versus in (A/A_0) calculated from (c-g). Absorption intensity of BTIC-4Cl NPs (c), BTIC-OH-*δ* NPs (d), BTIC-*δ*OH-2Cl NPs (e), ICG (f), and Ce6 (g) mixed with DPBF under laser irradiation over time (The Ce6 group was irradiated by 660 nm laser, 0.6 W cm^{-2} and the other groups were irradiated by 808 nm laser, 0.6 W cm^{-2}).

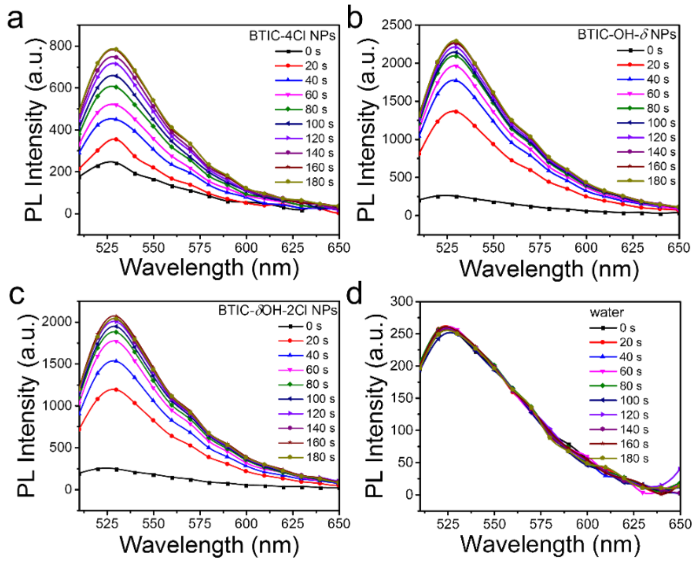

Figure S19. PL intensity of BTIC-4Cl NPs (a), BTIC-OH-*δ* NPs (b), BTIC-*δ*OH-2Cl NPs (c), and water (d) mixed with SOSG under power density (808 nm laser, 0.6 W cm^{-2}) over time.

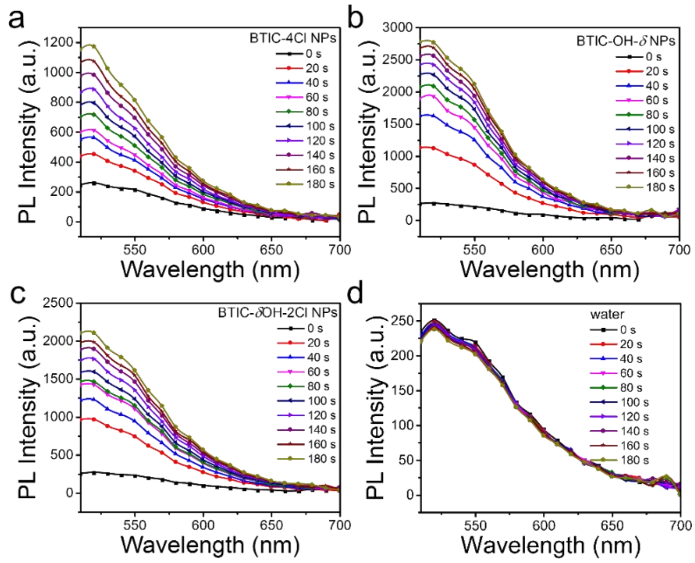

Figure S20. PL intensity of BTIC-4Cl NPs (a), BTIC-OH-*δ* NPs (b), BTIC-*δ*OH-2Cl NPs (c), and water (d) mixed with APF under power density (808 nm laser, 0.6 W cm^{-2}) over time.

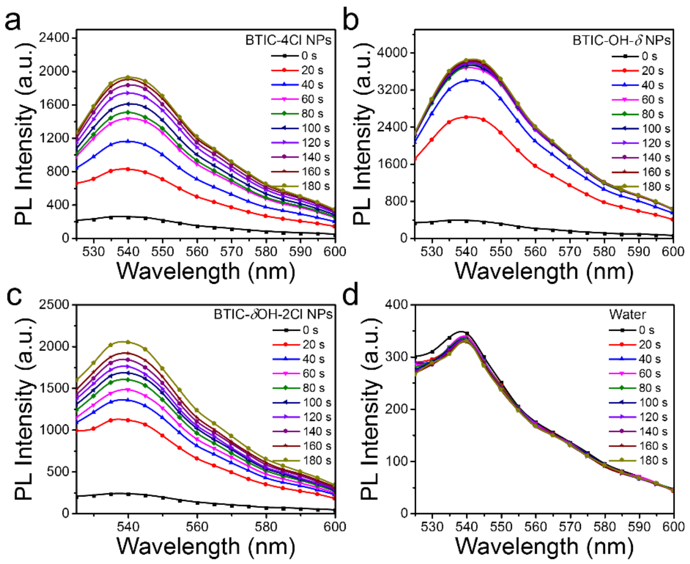

Figure S21. PL intensity of BTIC-4Cl NPs (a), BTIC-OH-δ NPs (b), BTIC-δOH-2Cl NPs (c), and water (d) mixed with DHR 123 under power density (808 nm laser, 0.6 W cm^{-2}) over time.

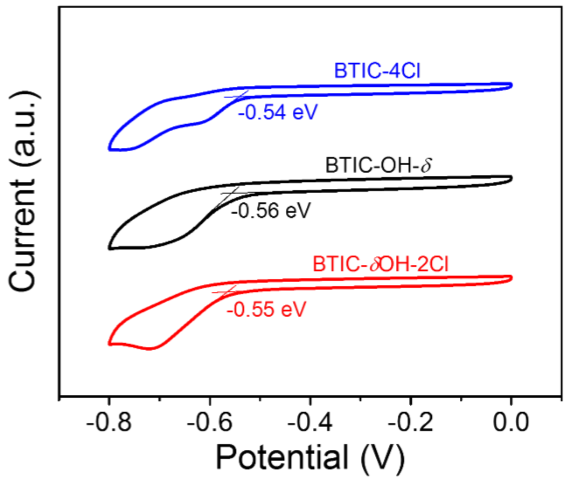

Figure S22. The cyclic voltammetry of BTIC-OH-δ, BTIC-δOH-2Cl, and BTIC-4Cl.

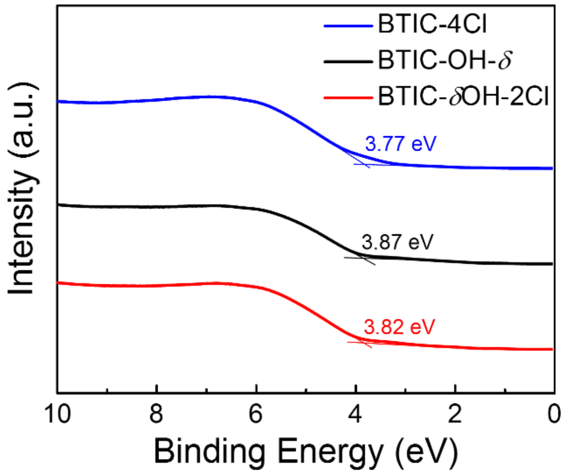

Figure S23. The XPS valence band spectrum of BTIC-OH-*δ*, BTIC-*δ*OH-2Cl, and BTIC-4Cl.

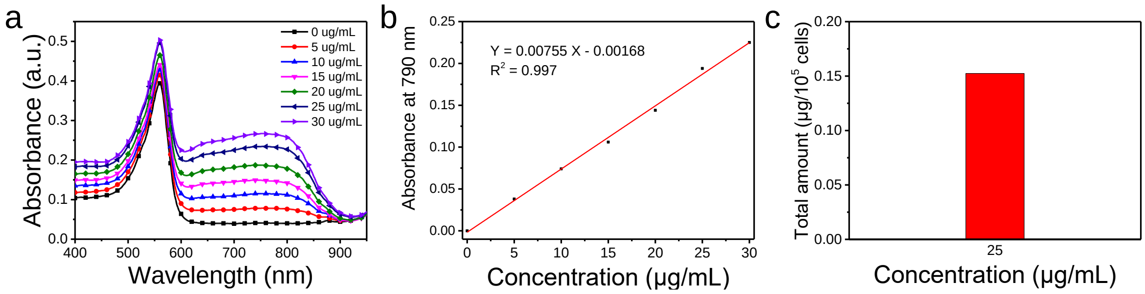

Figure S24. (a-b) The absorbance spectrum of BTIC-*δ*OH-2Cl NPs in the cell culture media as a function of concentration. (c) Internalized amounts of BTIC-*δ*OH-2Cl NPs in 4T1 cells after 24 h incubation with 30 μg mL^{-1} concentrations of the sample.

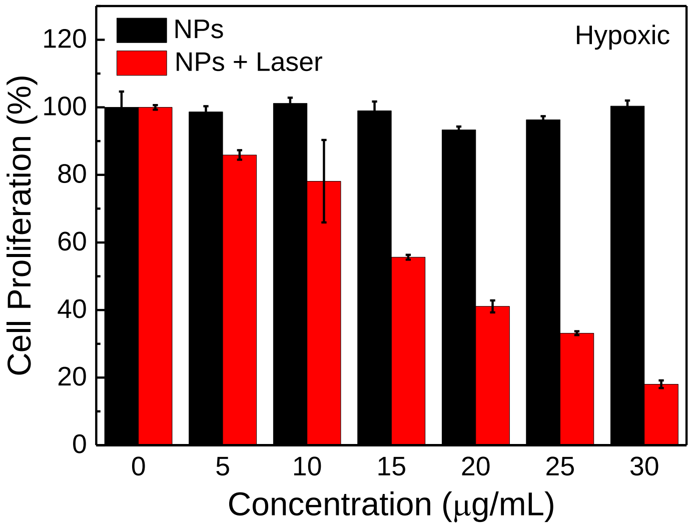

Figure S25. Cell proliferation of 4T1 cells incubated with BTIC-δOH-2Cl NPs at various concentrations in the dark and after 360 J cm^{-2} NIR light irradiation under hypoxic conditions (808 nm, 0.6 W cm^{-2} for 10 min).

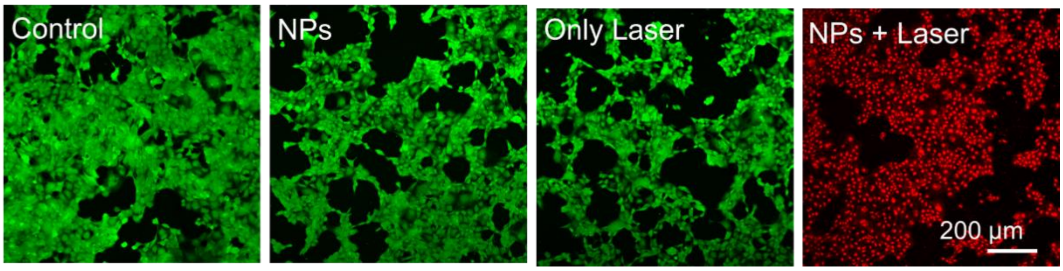

Figure S26. CLSM images of calcein AM/PI-stained 4T1 cells. NIR light irradiation (808 nm, 0.6 W cm^{-2}, 10 min) was conducted after cells were incubated with BTIC-δOH-2Cl NPs (15 μg mL^{-1}).

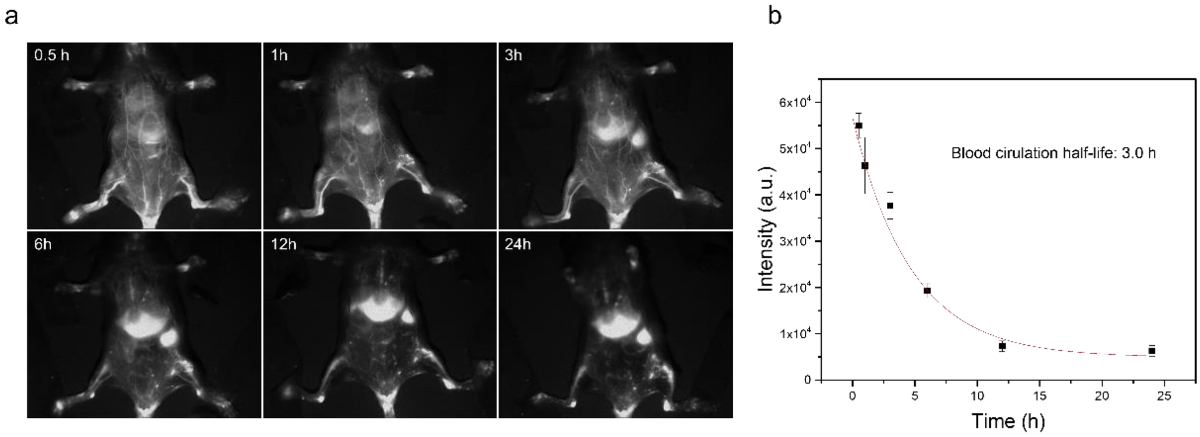

Figure S27. (a) BALB/c mice were tail-vein injected with BTIC-δOH-2Cl NPs to study the clearance of BTIC-δOH-2Cl NPs from mouse blood circulatory system. (b) The signal intensity of mice femoral artery as a function of time.[5] Error bars are the standard deviation of the mean (n=3).

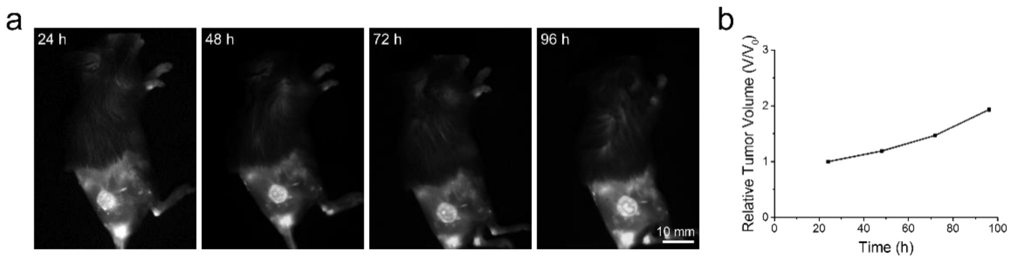

Figure S28. Tumor growth trend monitored by in vivo NIR-II FLI. (a) In vivo NIR-II FLI of tumors after intravenous injection of BTIC-δOH-2Cl NPs at different time intervals in 4T1 tumor-bearing mice. (b) Tumor growth profile of 4T1 tumor-bearing mice.

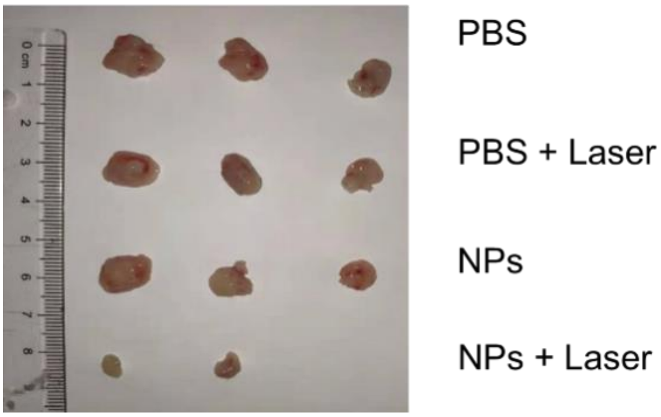

Figure S29. Pictures of the tumors collected 14 days after with different treatments.

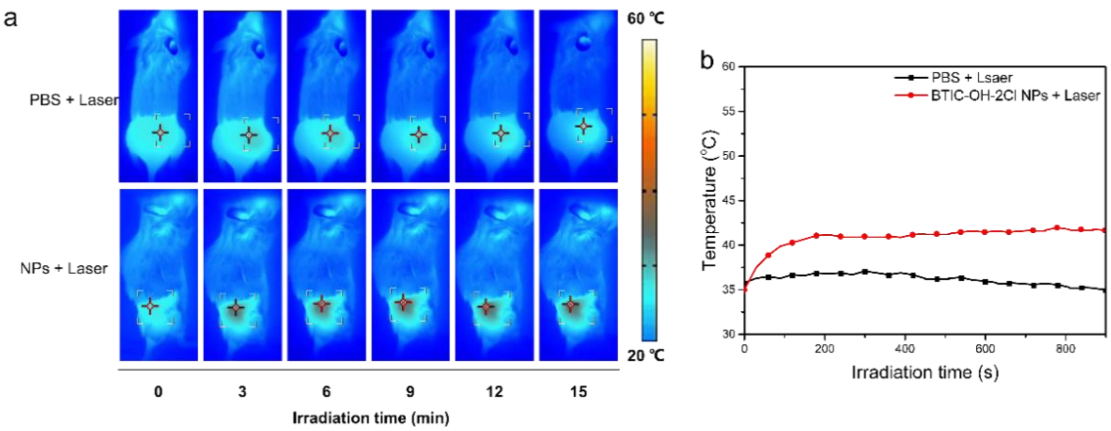

Figure S30. (a) IR thermal images of the 4T1 tumor-bearing mice treated with PBS + laser and BTIC-δOH-2Cl NPs + laser group (0.5 mg mL^{-1}, 0.2 mL; 0.6 W cm^{-2}, 808 nm laser). (b) Corresponding temperature profiles of tumors in mice with different laser treatments.

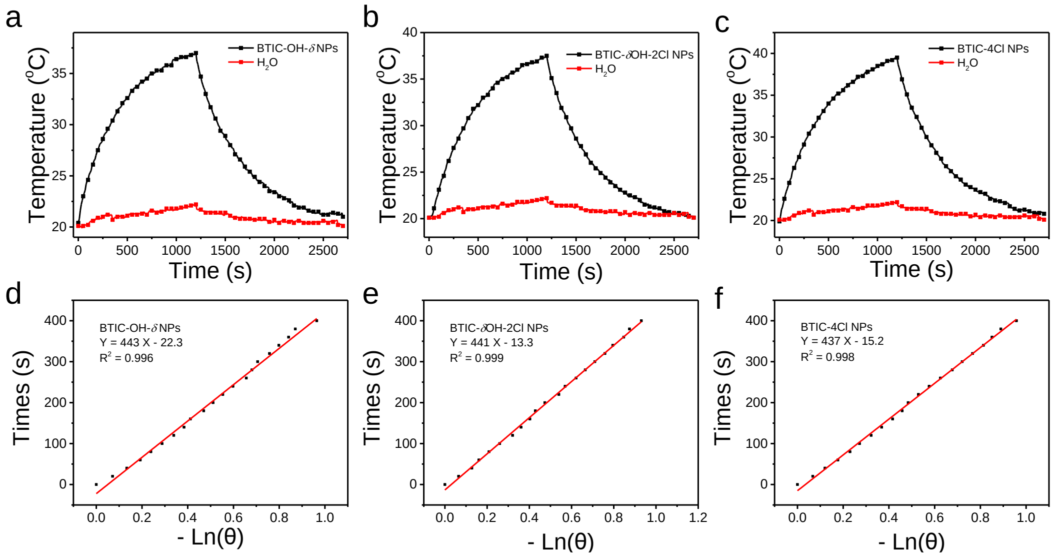

Figure S31. Data in photothermal conversion efficiency (PCE) calculation. Temperature elevation curves under 808 nm irradiation at 0.8 W for 1200 s, followed by subsequent cooling to room temperature and plots of time against the negative natural logarithm of temperature variation during the cooling processes. BTIC-OH-δ (a, d), BTIC-δOH-2Cl (b, e), and BTIC-4Cl (c, f) NPs were calculated to be 48 %, 52 %, and 55% in water.

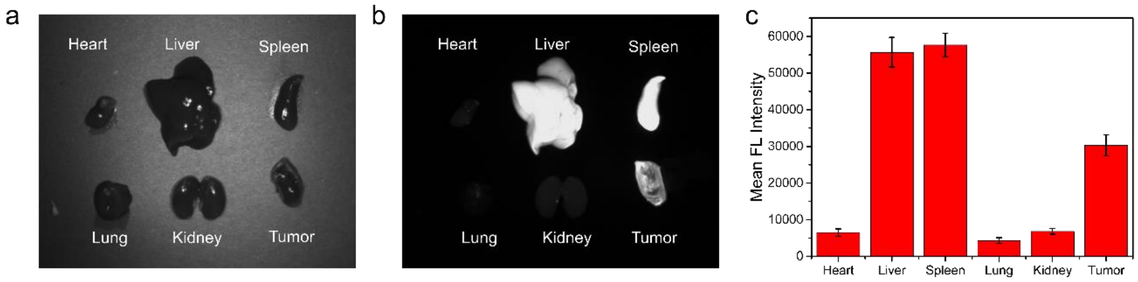

Figure S32. BTIC-δOH-2Cl NPs in the main organs and tumor tissues of mice at 24 h post intravenous administration. (a) Photo under halogen lamp and (b) fluorescence images of major organs and the tumor at 24 h post intravenous administration (heart, liver, spleen, lung, kidneys, and tumor). (c) The signal intensity of major organs and the tumor in (b). Error bars are the standard deviation of the mean (n=3).

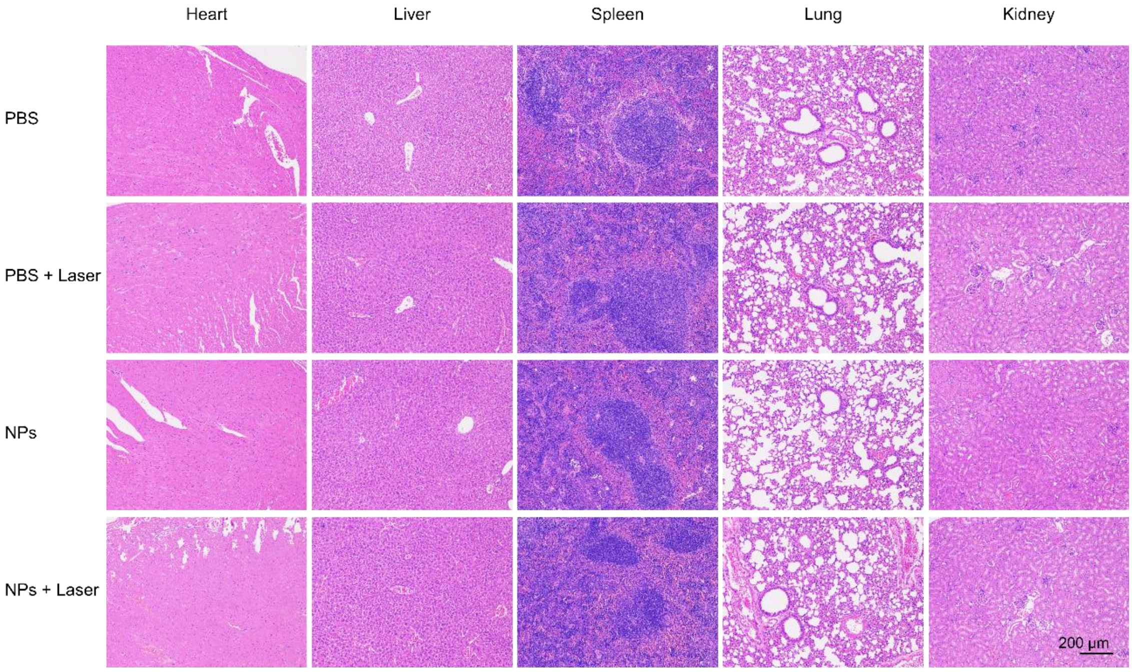

Figure S33. Histological staining (Hematoxylin and Eosin Staining) analysis of major organs collected from the mice after 14-day treatments.

Table S1. Blood tests for mice in different treatment groups after 14-day treatments.

	White blood cells (K μL^{-1})	Central mitochondrial cell (K μL^{-1})	Lymphocyte cell (K μL^{-1})
PBS	86.6	69.1	10.5
PBS + Laser	85.3	66.4	7.1
NPs	89.2	69.9	11.1
NPs + Laser	6.06	1.07	4.8
Health	4.75	1.93	2.5

References

[1] H. Lai, L. Liu, N. Zheng, L. Han, F. He, *J. Phys. Chem. Lett.* **2021**, *12*, 4666-4673.

[2] M. J. Frisch, G. W. Trucks, H. B. Schlegel, G. E. Scuseria, M. A. Robb, J. R. Cheeseman, G. Scalmani, V. Barone, B. Mennucci, G. A. Petersson, H. Nakatsuji, M. Caricato, X. Li, H. P. Hratchian, A. F. Izmaylov, J. Bloino, G. Zheng, J. L. Sonnenberg, M. Hada, M. Ehara, K. Toyota, R. Fukuda, J. Hasegawa, M. Ishida, T. Nakajima, Y. Honda, O. Kitao, H. Nakai, T. Vreven, J. A. Montgomery, Jr., J. E. Peralta, F. Ogliaro, M. Bearpark, J. J. Heyd, E. Brothers, K. N. Kudin, V. N. Staroverov, R. Kobayashi, J. Normand, K. Raghavachari, A.

Rendell, J. C. Burant, S. S. Iyengar, J. Tomasi, M. Cossi, N. Rega, J. M. Millam, M. Klene, J. E. Knox, J. B. Cross, V. Bakken, C. Adamo, J. Jaramillo, R. Gomperts, R. E. Stratmann, O. Yazyev, A. J. Austin, R. Cammi, C. Pomelli, J. W. Ochterski, R. L. Martin, K. Morokuma, V. G. Zakrzewski, G. A. Voth, P. Salvador, J. J. Dannenberg, S. Dapprich, A. D. Daniels, Ö. Farkas, J. B. Foresman, J. V. Ortiz, J. Cioslowski, and D. J. Fox, *Gaussian 09, Revision A.1*, Gaussian, Inc., Wallingford CT, 2009.

[3] L. Li, C. Shao, T. Liu, Z. Chao, H. Chen, F. Xiao, H. He, Z. Wei, Y. Zhu, H. Wang, X. Zhang, Y. Wen, B. Yang, F. He, L. Tian, *Adv. Mater.* **2020**, *32*, 2003471.

[4] Y. Dai, J. Su, K. Wu, W. Ma, B. Wang, M. Li, P. Sun, Q. Shen, Q. Wang, Q. Fan, *ACS Appl. Mater. Interfaces* **2019**, *11*, 10540.

[5] Y. Zhong, Z. Ma, F. Wang, X. Wang, Y. Yang, Y. Liu, X. Zhao, J. Li, H. Du, M. Zhang, Q. Cui, S. Zhu, Q. Sun, H. Wan, Y. Tian, Q. Liu, W. Wang, K. C. Garcia, H. Dai, *Nat. Biotechnol.* **2019**, *37*, 1322.
