# Supplementary material for: The Balance Effect of π–π Electronic Coupling on NIR‐II Emission and Photodynamic Properties of Highly Hydrophobic Conjugated Photosensitizers
Source: Adv Sci (Weinh). 2023 Dec 28;11(6):2307569. doi: 10.1002/advs.202307569 (PMC10853711; doi:10.1002/advs.202307569)

checkCIF/PLATON report

Structure factors have been supplied for datablock(s) 5oh-3_sq

THIS REPORT IS FOR GUIDANCE ONLY. IF USED AS PART OF A REVIEW PROCEDURE FOR PUBLICATION, IT SHOULD NOT REPLACE THE EXPERTISE OF AN EXPERIENCED CRYSTALLOGRAPHIC REFEREE.

No syntax errors found. CIF dictionary Interpreting this report

Datablock: 5oh-3_sq

Bond precision:	C-C = 0.0273 A	Wavelength=1.54178

Cell:	a=23.712(2)	b=33.048(3)	c=20.8658(18)
	alpha=90	beta=94.880(4)	gamma=90
Temperature:	102 K		

	Calculated	Reported
Volume	16292(2)	16292(3)
Space group	C 2/c	C 1 2/c 1
Hall group	-C 2yc	-C 2yc
Moiety formula	C64 H10 N8 O4 S5, 2(O), C [+ solvent]	C65 H10 N8 O4 S5, 2(O)
Sum formula	C65 H10 N8 O6 S5 [+ solvent]	C65 H10 N8 O6 S5
Mr	1159.11	1159.11
Dx,g cm-3	0.945	0.945
Z	8	8
Mu (mm-1)	1.665	1.665
F000	4672.0	4672.0
F000’	4698.38	
h,k,lmax	19,27,17	19,27,17
Nref	4883	4846
Tmin,Tmax		
Tmin’		

Correction method= Not given

Data completeness= 0.992 Theta(max)= 39.760

R(reflections)= 0.1206(3499) wR2(reflections)= 0.3774(4846)

S = 0.938 Npar= 718

The following ALERTS were generated. Each ALERT has the format
test-name_ALERT_alert-type_alert-level.
Click on the hyperlinks for more details of the test.

Alert level A

```
THETM01_ALERT_3_A  The value of sine(theta_max)/wavelength is less than 0.550
            Calculated sin(theta_max)/wavelength =    0.4148
```

Alert level B

```
PLAT084_ALERT_3_B High wR2 Value (i.e. > 0.25) ...................       0.38 Report
PLAT088_ALERT_3_B Poor Data / Parameter Ratio ....................       6.75 Note
PLAT234_ALERT_4_B Large Hirshfeld Difference N8       --C54      .       0.26 Ang.
PLAT234_ALERT_4_B Large Hirshfeld Difference N9       --C48      .       0.26 Ang.
PLAT234_ALERT_4_B Large Hirshfeld Difference C46      --C47      .       0.30 Ang.
PLAT234_ALERT_4_B Large Hirshfeld Difference C52      --C53      .       0.30 Ang.
PLAT234_ALERT_4_B Large Hirshfeld Difference C63      --C64      .       0.26 Ang.
PLAT260_ALERT_2_B Large Average Ueq of Residue Including        O5      0.383 Check
PLAT260_ALERT_2_B Large Average Ueq of Residue Including      C1AA      0.367 Check
PLAT306_ALERT_2_B Isolated Oxygen Atom (H-atoms Missing ?) .......       O0AA Check
PLAT306_ALERT_2_B Isolated Oxygen Atom (H-atoms Missing ?) .......         O5 Check
PLAT315_ALERT_2_B Singly Bonded Carbon Detected (H-atoms Missing).        C44 Check
PLAT315_ALERT_2_B Singly Bonded Carbon Detected (H-atoms Missing).        C51 Check
PLAT315_ALERT_2_B Singly Bonded Carbon Detected (H-atoms Missing).        C53 Check
PLAT315_ALERT_2_B Singly Bonded Carbon Detected (H-atoms Missing).        C56 Check
PLAT315_ALERT_2_B Singly Bonded Carbon Detected (H-atoms Missing).        C59 Check
PLAT315_ALERT_2_B Singly Bonded Carbon Detected (H-atoms Missing).        C64 Check
PLAT340_ALERT_3_B Low Bond Precision on  C-C Bonds ...............    0.02726 Ang.
PLAT420_ALERT_2_B D-H Bond Without Acceptor  O1       --H1       .     Please Check
```

Alert level C

```
PLAT052_ALERT_1_C Info on Absorption Correction Method   Not Given     Please Do !
PLAT053_ALERT_1_C Minimum Crystal Dimension Missing (or Error) ...     Please Check
PLAT054_ALERT_1_C Medium  Crystal Dimension Missing (or Error) ...     Please Check
PLAT055_ALERT_1_C Maximum Crystal Dimension Missing (or Error) ...     Please Check
PLAT082_ALERT_2_C High R1 Value ..................................       0.12 Report
PLAT094_ALERT_2_C Ratio of Maximum / Minimum Residual Density ....       2.30 Report
PLAT202_ALERT_3_C Isotropic non-H Atoms in Anion/Solvent .........          1 Check
             O0AA
PLAT220_ALERT_2_C NonSolvent   Resd 1  C   Ueq(max)/Ueq(min) Range         3.3 Ratio
PLAT234_ALERT_4_C Large Hirshfeld Difference S2       --C28      .       0.21 Ang.
PLAT234_ALERT_4_C Large Hirshfeld Difference S3       --N6       .       0.21 Ang.
PLAT234_ALERT_4_C Large Hirshfeld Difference N7       --C22      .       0.21 Ang.
PLAT234_ALERT_4_C Large Hirshfeld Difference C6       --C10      .       0.24 Ang.
PLAT234_ALERT_4_C Large Hirshfeld Difference C13      --C14      .       0.21 Ang.
PLAT234_ALERT_4_C Large Hirshfeld Difference C15      --C16      .       0.20 Ang.
PLAT234_ALERT_4_C Large Hirshfeld Difference C21      --C25      .       0.19 Ang.
PLAT234_ALERT_4_C Large Hirshfeld Difference C22      --C23      .       0.23 Ang.
PLAT234_ALERT_4_C Large Hirshfeld Difference C48      --C49      .       0.22 Ang.
PLAT234_ALERT_4_C Large Hirshfeld Difference C49      --C50      .       0.22 Ang.
PLAT234_ALERT_4_C Large Hirshfeld Difference C58      --C59      .       0.22 Ang.
PLAT241_ALERT_2_C High   'MainMol' Ueq as Compared to Neighbors of         S1 Check
PLAT241_ALERT_2_C High   'MainMol' Ueq as Compared to Neighbors of         S3 Check
```

```
PLAT241_ALERT_2_C High   'MainMol' Ueq as Compared to Neighbors of         S4 Check
PLAT241_ALERT_2_C High   'MainMol' Ueq as Compared to Neighbors of        C46 Check
PLAT242_ALERT_2_C Low    'MainMol' Ueq as Compared to Neighbors of         C1 Check
PLAT242_ALERT_2_C Low    'MainMol' Ueq as Compared to Neighbors of         C3 Check
PLAT242_ALERT_2_C Low    'MainMol' Ueq as Compared to Neighbors of         C8 Check
PLAT242_ALERT_2_C Low    'MainMol' Ueq as Compared to Neighbors of         C9 Check
PLAT242_ALERT_2_C Low    'MainMol' Ueq as Compared to Neighbors of        C36 Check
PLAT242_ALERT_2_C Low    'MainMol' Ueq as Compared to Neighbors of        C40 Check
PLAT242_ALERT_2_C Low    'MainMol' Ueq as Compared to Neighbors of        C43 Check
PLAT242_ALERT_2_C Low    'MainMol' Ueq as Compared to Neighbors of        C47 Check
PLAT242_ALERT_2_C Low    'MainMol' Ueq as Compared to Neighbors of        C49 Check
PLAT242_ALERT_2_C Low    'MainMol' Ueq as Compared to Neighbors of        C50 Check
PLAT242_ALERT_2_C Low    'MainMol' Ueq as Compared to Neighbors of        C54 Check
PLAT242_ALERT_2_C Low    'MainMol' Ueq as Compared to Neighbors of        C60 Check
PLAT242_ALERT_2_C Low    'MainMol' Ueq as Compared to Neighbors of        C62 Check
PLAT242_ALERT_2_C Low    'MainMol' Ueq as Compared to Neighbors of        C63 Check
PLAT242_ALERT_2_C Low    'MainMol' Ueq as Compared to Neighbors of        C82 Check
PLAT260_ALERT_2_C Large Average Ueq of Residue Including       S1      0.205 Check
PLAT911_ALERT_3_C Missing FCF Refl Between Thmin & STh/L=    0.415         35 Report
PLAT918_ALERT_3_C Reflection(s) with I(obs) much Smaller I(calc) .         11 Check
```

Alert level G

```
PLAT002_ALERT_2_G Number of Distance or Angle Restraints on AtSite         41 Note
PLAT003_ALERT_2_G Number of Uiso or Uij Restrained non-H Atoms ...         82 Report
PLAT007_ALERT_5_G Number of Unrefined Donor-H Atoms .............           2 Report
PLAT042_ALERT_1_G Calc. and Reported MoietyFormula Strings  Differ     Please Check
PLAT072_ALERT_2_G SHELXL First  Parameter in WGHT  Unusually Large       0.25 Report
PLAT083_ALERT_2_G SHELXL Second Parameter in WGHT  Unusually Large     100.00 Why ?
PLAT172_ALERT_4_G The CIF-Embedded .res File Contains DFIX Records         12 Report
PLAT173_ALERT_4_G The CIF-Embedded .res File Contains DANG Records         12 Report
PLAT176_ALERT_4_G The CIF-Embedded .res File Contains SADI Records          2 Report
PLAT178_ALERT_4_G The CIF-Embedded .res File Contains SIMU Records          1 Report
PLAT343_ALERT_2_G Unusual sp?      Angle Range in Main Residue for        C43 Check
PLAT343_ALERT_2_G Unusual sp?      Angle Range in Main Residue for        C44 Check
PLAT343_ALERT_2_G Unusual sp?      Angle Range in Main Residue for        C46 Check
PLAT343_ALERT_2_G Unusual sp?      Angle Range in Main Residue for        C47 Check
PLAT343_ALERT_2_G Unusual sp?      Angle Range in Main Residue for        C48 Check
PLAT343_ALERT_2_G Unusual sp?      Angle Range in Main Residue for        C49 Check
PLAT343_ALERT_2_G Unusual sp?      Angle Range in Main Residue for        C50 Check
PLAT343_ALERT_2_G Unusual sp?      Angle Range in Main Residue for        C51 Check
PLAT343_ALERT_2_G Unusual sp?      Angle Range in Main Residue for        C52 Check
PLAT343_ALERT_2_G Unusual sp?      Angle Range in Main Residue for        C53 Check
PLAT343_ALERT_2_G Unusual sp?      Angle Range in Main Residue for        C54 Check
PLAT343_ALERT_2_G Unusual sp?      Angle Range in Main Residue for        C55 Check
PLAT343_ALERT_2_G Unusual sp?      Angle Range in Main Residue for        C56 Check
PLAT343_ALERT_2_G Unusual sp?      Angle Range in Main Residue for        C57 Check
PLAT343_ALERT_2_G Unusual sp?      Angle Range in Main Residue for        C58 Check
PLAT343_ALERT_2_G Unusual sp?      Angle Range in Main Residue for        C59 Check
PLAT343_ALERT_2_G Unusual sp?      Angle Range in Main Residue for        C62 Check
PLAT343_ALERT_2_G Unusual sp?      Angle Range in Main Residue for        C63 Check
PLAT343_ALERT_2_G Unusual sp?      Angle Range in Main Residue for        C64 Check
PLAT343_ALERT_2_G Unusual sp?      Angle Range in Main Residue for        C80 Check
PLAT344_ALERT_2_G Unusual sp?       Angle Range in Solvent/Ion for       C1AA Check
PLAT367_ALERT_2_G Long?  C(sp?)-C(sp?) Bond  C14      - C80      .       1.56 Ang.
PLAT367_ALERT_2_G Long?  C(sp?)-C(sp?) Bond  C30      - C47      .       1.57 Ang.
PLAT367_ALERT_2_G Long?  C(sp?)-C(sp?) Bond  C43      - C44      .       1.56 Ang.
```

```
PLAT367_ALERT_2_G Long?  C(sp?)-C(sp?) Bond  C43      - C46      .       1.60 Ang.
PLAT367_ALERT_2_G Long?  C(sp?)-C(sp?) Bond  C46      - C47      .       1.51 Ang.
PLAT367_ALERT_2_G Long?  C(sp?)-C(sp?) Bond  C48      - C49      .       1.62 Ang.
PLAT367_ALERT_2_G Long?  C(sp?)-C(sp?) Bond  C49      - C52      .       1.54 Ang.
PLAT367_ALERT_2_G Long?  C(sp?)-C(sp?) Bond  C50      - C51      .       1.58 Ang.
PLAT367_ALERT_2_G Long?  C(sp?)-C(sp?) Bond  C52      - C53      .       1.58 Ang.
PLAT367_ALERT_2_G Long?  C(sp?)-C(sp?) Bond  C54      - C55      .       1.66 Ang.
PLAT367_ALERT_2_G Long?  C(sp?)-C(sp?) Bond  C55      - C57      .       1.53 Ang.
PLAT367_ALERT_2_G Long?  C(sp?)-C(sp?) Bond  C55      - C58      .       1.64 Ang.
PLAT367_ALERT_2_G Long?  C(sp?)-C(sp?) Bond  C56      - C57      .       1.68 Ang.
PLAT367_ALERT_2_G Long?  C(sp?)-C(sp?) Bond  C58      - C59      .       1.62 Ang.
PLAT367_ALERT_2_G Long?  C(sp?)-C(sp?) Bond  C62      - C63      .       1.65 Ang.
PLAT367_ALERT_2_G Long?  C(sp?)-C(sp?) Bond  C62      - C80      .       1.56 Ang.
PLAT432_ALERT_2_G Short Inter X...Y Contact  C1AA     ..C59      .       1.81 Ang.
                                                      x,y,z  =     1_555 Check
PLAT432_ALERT_2_G Short Inter X...Y Contact  C1AA     ..C58      .       2.93 Ang.
                                                      x,y,z  =     1_555 Check
PLAT606_ALERT_4_G Solvent Accessible VOID(S) in Structure ........          ! Info
PLAT720_ALERT_4_G Number of Unusual/Non-Standard Labels ..........          3 Note
PLAT773_ALERT_2_G Check long C-C Bond in CIF: C59      --C1AA            1.81 Ang.
PLAT790_ALERT_4_G Centre of Gravity not Within Unit Cell: Resd.  #          2 Note
              O
PLAT860_ALERT_3_G Number of Least-Squares Restraints .............        627 Note
PLAT869_ALERT_4_G ALERTS Related to the Use of SQUEEZE  Suppressed          ! Info
PLAT883_ALERT_1_G No Info/Value for _atom_sites_solution_primary .     Please Do !
PLAT909_ALERT_3_G Percentage of I>2sig(I) Data at Theta(Max) Still        47% Note
PLAT910_ALERT_3_G Missing # of FCF Reflection(s) Below Theta(Min).          2 Note
PLAT913_ALERT_3_G Missing # of Very Strong Reflections in FCF ....          2 Note
PLAT978_ALERT_2_G Number C-C Bonds with Positive Residual Density.          0 Info
```

---

1 **ALERT level A** = Most likely a serious problem - resolve or explain
19 **ALERT level B** = A potentially serious problem, consider carefully
41 **ALERT level C** = Check. Ensure it is not caused by an omission or oversight
60 **ALERT level G** = General information/check it is not something unexpected

6 ALERT type 1 CIF construction/syntax error, inconsistent or missing data
79 ALERT type 2 Indicator that the structure model may be wrong or deficient
11 ALERT type 3 Indicator that the structure quality may be low
24 ALERT type 4 Improvement, methodology, query or suggestion
1 ALERT type 5 Informative message, check

It is advisable to attempt to resolve as many as possible of the alerts in all categories. Often the minor alerts point to easily fixed oversights, errors and omissions in your CIF or refinement strategy, so attention to these fine details can be worthwhile. In order to resolve some of the more serious problems it may be necessary to carry out additional measurements or structure refinements. However, the purpose of your study may justify the reported deviations and the more serious of these should normally be commented upon in the discussion or experimental section of a paper or in the "special_details" fields of the CIF. checkCIF was carefully designed to identify outliers and unusual parameters, but every test has its limitations and alerts that are not important in a particular case may appear. Conversely, the absence of alerts does not guarantee there are no aspects of the results needing attention. It is up to the individual to critically assess their own results and, if necessary, seek expert advice.

Publication of your CIF in IUCr journals

A basic structural check has been run on your CIF. These basic checks will be run on all CIFs submitted for publication in IUCr journals (*Acta Crystallographica*, *Journal of Applied Crystallography*, *Journal of Synchrotron Radiation*); however, if you intend to submit to *Acta Crystallographica Section C* or *E* or *IUCrData*, you should make sure that full publication checks are run on the final version of your CIF prior to submission.

Publication of your CIF in other journals

Please refer to the *Notes for Authors* of the relevant journal for any special instructions relating to CIF submission.

PLATON version of 18/05/2022; check.def file version of 17/05/2022

Datablock 5oh-3_sq - ellipsoid plot

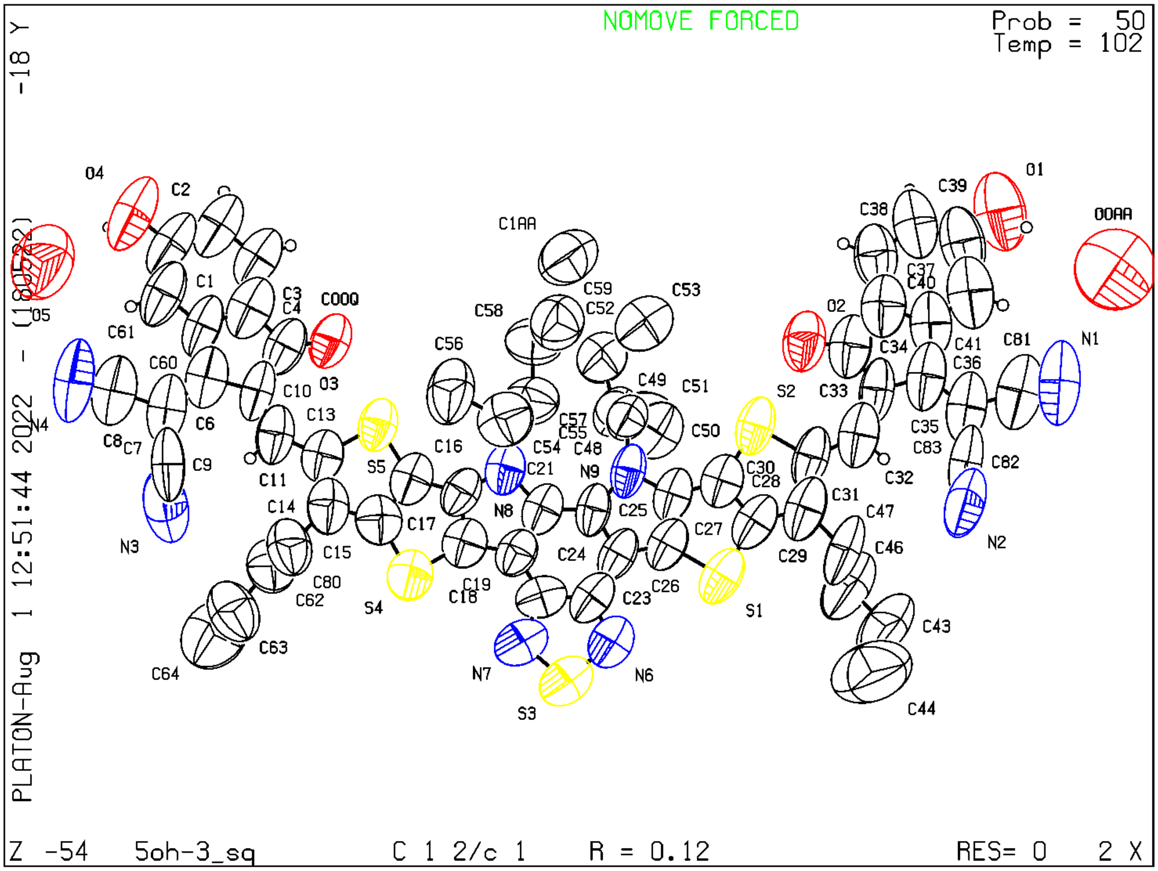

Supplement: Supplementary file 2 — Supporting Information [file ADVS-11-2307569-s002.zip › advs202307569-sup-0002-cif/BTIC-2OH-checkcif.pdf]
