# Supplementary material for: The Balance Effect of π–π Electronic Coupling on NIR‐II Emission and Photodynamic Properties of Highly Hydrophobic Conjugated Photosensitizers
Source: Adv Sci (Weinh). 2023 Dec 28;11(6):2307569. doi: 10.1002/advs.202307569 (PMC10853711; doi:10.1002/advs.202307569)

checkCIF/PLATON report

Structure factors have been supplied for datablock(s) y7-2_sq

THIS REPORT IS FOR GUIDANCE ONLY. IF USED AS PART OF A REVIEW PROCEDURE FOR PUBLICATION, IT SHOULD NOT REPLACE THE EXPERTISE OF AN EXPERIENCED CRYSTALLOGRAPHIC REFEREE.

No syntax errors found. CIF dictionary Interpreting this report

Datablock: y7-2_sq

Bond precision: C-C = 0.0478 A Wavelength=1.54178

Cell:	a=24.082(2)	b=57.855(6)	c=13.8091(11)
	alpha=90	beta=104.745(4)	gamma=90
Temperature:	103 K		

	Calculated	Reported
Volume	18606(3)	18606(3)
Space group	C 2/c	C 1 2/c 1
Hall group	-C 2yc	-C 2yc
Moiety formula	2(C62 H6 Cl4 N8 O2 S5), C Br2, 2(O) [+ solvent]	C62 H6 Cl4 N8 O2 S5, 0.5(Br2 C), O
Sum formula	C125 H12 Br2 Cl8 N16 O6 S10 [+ solvent]	C62.50 H6 Br Cl4 N8 O3 S5
Mr	2597.51	1298.76
Dx,g cm-3	0.927	0.927
Z	4	8
Mu (mm-1)	2.990	2.990
F000	5152.0	5152.0
F000'	5183.34	
h,k,lmax	18,45,10	18,45,10
Nref	4646	4629
Tmin,Tmax		
Tmin'		

Correction method= Not given

Data completeness= 0.996 Theta(max)= 36.998

R(reflections)= 0.1843(3689) wR2(reflections)= 0.4497(4629)

S = 0.935 Npar= 728

The following ALERTS were generated. Each ALERT has the format
test-name_ALERT_alert-type_alert-level.
Click on the hyperlinks for more details of the test.

Alert level A

```
THETM01_ALERT_3_A  The value of sine(theta_max)/wavelength is less than 0.550
            Calculated sin(theta_max)/wavelength =    0.3903
```

Alert level B

```
PLAT082_ALERT_2_B High R1 Value ..................................       0.18 Report
PLAT084_ALERT_3_B High wR2 Value (i.e. > 0.25) ...................       0.45 Report
PLAT088_ALERT_3_B Poor Data / Parameter Ratio ....................       6.36 Note
PLAT234_ALERT_4_B Large Hirshfeld Difference C15      --C6       .       0.26 Ang.
PLAT234_ALERT_4_B Large Hirshfeld Difference C40      --C54      .       0.30 Ang.
PLAT306_ALERT_2_B Isolated Oxygen Atom (H-atoms Missing ?) .......         O2 Check
PLAT315_ALERT_2_B Singly Bonded Carbon Detected (H-atoms Missing).        C40 Check
PLAT315_ALERT_2_B Singly Bonded Carbon Detected (H-atoms Missing).        C43 Check
PLAT315_ALERT_2_B Singly Bonded Carbon Detected (H-atoms Missing).        C48 Check
PLAT315_ALERT_2_B Singly Bonded Carbon Detected (H-atoms Missing).        C52 Check
PLAT315_ALERT_2_B Singly Bonded Carbon Detected (H-atoms Missing).        C53 Check
PLAT315_ALERT_2_B Singly Bonded Carbon Detected (H-atoms Missing).        C58 Check
PLAT341_ALERT_3_B Low Bond Precision on  C-C Bonds ...............    0.04781 Ang.
PLAT430_ALERT_2_B Short Inter D...A Contact  O2        ..O2       .       2.61 Ang.
                                             1-x,y,1/2-z  =      2_655 Check
```

Alert level C

```
PLAT053_ALERT_1_C Minimum Crystal Dimension Missing (or Error) ...     Please Check
PLAT054_ALERT_1_C Medium  Crystal Dimension Missing (or Error) ...     Please Check
PLAT055_ALERT_1_C Maximum Crystal Dimension Missing (or Error) ...     Please Check
PLAT094_ALERT_2_C Ratio of Maximum / Minimum Residual Density ....       3.09 Report
PLAT202_ALERT_3_C Isotropic non-H Atoms in Anion/Solvent .........          1 Check
              O2
PLAT213_ALERT_2_C Atom C52               has ADP max/min Ratio .....      3.1 prolat
PLAT220_ALERT_2_C NonSolvent   Resd 1   C   Ueq(max)/Ueq(min) Range       3.8 Ratio
PLAT234_ALERT_4_C Large Hirshfeld Difference N20      --C46      .       0.22 Ang.
PLAT234_ALERT_4_C Large Hirshfeld Difference C38      --C98      .       0.22 Ang.
PLAT241_ALERT_2_C High   'MainMol' Ueq as Compared to Neighbors of         S1 Check
PLAT241_ALERT_2_C High   'MainMol' Ueq as Compared to Neighbors of         S3 Check
PLAT241_ALERT_2_C High   'MainMol' Ueq as Compared to Neighbors of         S4 Check
PLAT241_ALERT_2_C High   'MainMol' Ueq as Compared to Neighbors of         S5 Check
PLAT241_ALERT_2_C High   'MainMol' Ueq as Compared to Neighbors of         C4 Check
PLAT241_ALERT_2_C High   'MainMol' Ueq as Compared to Neighbors of        C46 Check
PLAT241_ALERT_2_C High   'MainMol' Ueq as Compared to Neighbors of        C50 Check
PLAT242_ALERT_2_C Low    'MainMol' Ueq as Compared to Neighbors of         N9 Check
PLAT242_ALERT_2_C Low    'MainMol' Ueq as Compared to Neighbors of         C2 Check
PLAT242_ALERT_2_C Low    'MainMol' Ueq as Compared to Neighbors of         C5 Check
PLAT242_ALERT_2_C Low    'MainMol' Ueq as Compared to Neighbors of        C14 Check
PLAT242_ALERT_2_C Low    'MainMol' Ueq as Compared to Neighbors of        C15 Check
PLAT242_ALERT_2_C Low    'MainMol' Ueq as Compared to Neighbors of        C21 Check
PLAT242_ALERT_2_C Low    'MainMol' Ueq as Compared to Neighbors of        C22 Check
PLAT242_ALERT_2_C Low    'MainMol' Ueq as Compared to Neighbors of        C23 Check
PLAT242_ALERT_2_C Low    'MainMol' Ueq as Compared to Neighbors of        C26 Check
```

```
PLAT242_ALERT_2_C Low    'MainMol' Ueq as Compared to Neighbors of        C29 Check
PLAT242_ALERT_2_C Low    'MainMol' Ueq as Compared to Neighbors of        C39 Check
PLAT242_ALERT_2_C Low    'MainMol' Ueq as Compared to Neighbors of        C47 Check
PLAT242_ALERT_2_C Low    'MainMol' Ueq as Compared to Neighbors of        C57 Check
PLAT260_ALERT_2_C Large Average Ueq of Residue Including       Cl4      0.222 Check
PLAT260_ALERT_2_C Large Average Ueq of Residue Including       Br1      0.269 Check
PLAT906_ALERT_3_C Large K Value in the Analysis of Variance ......      6.351 Check
PLAT906_ALERT_3_C Large K Value in the Analysis of Variance ......      2.901 Check
PLAT911_ALERT_3_C Missing FCF Refl Between Thmin & STh/L=    0.390         15 Report
PLAT913_ALERT_3_C Missing # of Very Strong Reflections in FCF ....          5 Note
PLAT918_ALERT_3_C Reflection(s) with I(obs) much Smaller I(calc) .         19 Check
PLAT975_ALERT_2_C Check Calcd Resid. Dens.  0.95Ang From O2      .       1.17 eA-3
PLAT975_ALERT_2_C Check Calcd Resid. Dens.  0.97Ang From O2      .       1.02 eA-3
```

Alert level G

```
PLAT002_ALERT_2_G Number of Distance or Angle Restraints on AtSite         67 Note
PLAT003_ALERT_2_G Number of Uiso or Uij Restrained non-H Atoms ...         81 Report
PLAT042_ALERT_1_G Calc. and Reported MoietyFormula Strings  Differ     Please Check
PLAT045_ALERT_1_G Calculated and Reported Z Differ by a Factor ...      0.500 Check
PLAT072_ALERT_2_G SHELXL First  Parameter in WGHT  Unusually Large       0.20 Report
PLAT083_ALERT_2_G SHELXL Second Parameter in WGHT  Unusually Large     600.00 Why ?
PLAT172_ALERT_4_G The CIF-Embedded .res File Contains DFIX Records         19 Report
PLAT173_ALERT_4_G The CIF-Embedded .res File Contains DANG Records         18 Report
PLAT174_ALERT_4_G The CIF-Embedded .res File Contains FLAT Records          1 Report
PLAT176_ALERT_4_G The CIF-Embedded .res File Contains SADI Records          2 Report
PLAT177_ALERT_4_G The CIF-Embedded .res File Contains DELU Records          1 Report
PLAT178_ALERT_4_G The CIF-Embedded .res File Contains SIMU Records          5 Report
PLAT186_ALERT_4_G The CIF-Embedded .res File Contains ISOR Records          1 Report
PLAT300_ALERT_4_G Atom Site Occupancy of Br1        Constrained at        0.5 Check
PLAT300_ALERT_4_G Atom Site Occupancy of Br13       Constrained at        0.5 Check
PLAT300_ALERT_4_G Atom Site Occupancy of C13        Constrained at        0.5 Check
PLAT302_ALERT_4_G Anion/Solvent/Minor-Residue Disorder (Resd  2  )       100% Note
PLAT304_ALERT_4_G Non-Integer Number of Atoms in ..... (Resd  2  )       1.50 Check
PLAT343_ALERT_2_G Unusual sp?      Angle Range in Main Residue for         C1 Check
PLAT343_ALERT_2_G Unusual sp?      Angle Range in Main Residue for         C5 Check
PLAT343_ALERT_2_G Unusual sp?      Angle Range in Main Residue for        C18 Check
PLAT343_ALERT_2_G Unusual sp?      Angle Range in Main Residue for        C20 Check
PLAT343_ALERT_2_G Unusual sp?      Angle Range in Main Residue for        C29 Check
PLAT343_ALERT_2_G Unusual sp?      Angle Range in Main Residue for        C40 Check
PLAT343_ALERT_2_G Unusual sp?      Angle Range in Main Residue for        C43 Check
PLAT343_ALERT_2_G Unusual sp?      Angle Range in Main Residue for        C44 Check
PLAT343_ALERT_2_G Unusual sp?      Angle Range in Main Residue for        C45 Check
PLAT343_ALERT_2_G Unusual sp?      Angle Range in Main Residue for        C46 Check
PLAT343_ALERT_2_G Unusual sp?      Angle Range in Main Residue for        C47 Check
PLAT343_ALERT_2_G Unusual sp?      Angle Range in Main Residue for        C48 Check
PLAT343_ALERT_2_G Unusual sp?      Angle Range in Main Residue for        C49 Check
PLAT343_ALERT_2_G Unusual sp?      Angle Range in Main Residue for        C52 Check
PLAT343_ALERT_2_G Unusual sp?      Angle Range in Main Residue for        C53 Check
PLAT343_ALERT_2_G Unusual sp?      Angle Range in Main Residue for        C58 Check
PLAT343_ALERT_2_G Unusual sp?      Angle Range in Main Residue for        C60 Check
PLAT343_ALERT_2_G Unusual sp?      Angle Range in Main Residue for        C62 Check
PLAT367_ALERT_2_G Long?  C(sp?)-C(sp?) Bond  C1       - C5       .       1.54 Ang.
PLAT367_ALERT_2_G Long?  C(sp?)-C(sp?) Bond  C1       - C20      .       1.56 Ang.
PLAT367_ALERT_2_G Long?  C(sp?)-C(sp?) Bond  C1       - C29      .       1.54 Ang.
PLAT367_ALERT_2_G Long?  C(sp?)-C(sp?) Bond  C5       - C58      .       1.51 Ang.
PLAT367_ALERT_2_G Long?  C(sp?)-C(sp?) Bond  C18      - C43      .       1.54 Ang.
```

```
PLAT367_ALERT_2_G Long?  C(sp?)-C(sp?) Bond  C18      - C60      .       1.54 Ang.
PLAT367_ALERT_2_G Long?  C(sp?)-C(sp?) Bond  C20      - C45      .       1.54 Ang.
PLAT367_ALERT_2_G Long?  C(sp?)-C(sp?) Bond  C44      - C49      .       1.52 Ang.
PLAT367_ALERT_2_G Long?  C(sp?)-C(sp?) Bond  C44      - C53      .       1.61 Ang.
PLAT367_ALERT_2_G Long?  C(sp?)-C(sp?) Bond  C45      - C47      .       1.54 Ang.
PLAT367_ALERT_2_G Long?  C(sp?)-C(sp?) Bond  C46      - C60      .       1.56 Ang.
PLAT367_ALERT_2_G Long?  C(sp?)-C(sp?) Bond  C47      - C48      .       1.55 Ang.
PLAT367_ALERT_2_G Long?  C(sp?)-C(sp?) Bond  C49      - C62      .       1.53 Ang.
PLAT367_ALERT_2_G Long?  C(sp?)-C(sp?) Bond  C52      - C98      .       1.56 Ang.
PLAT367_ALERT_2_G Long?  C(sp?)-C(sp?) Bond  C60      - C62      .       1.54 Ang.
PLAT432_ALERT_2_G Short Inter X...Y Contact  N1       ..C13      .       2.94 Ang.
                                        -1/2+x,1/2-y,-1/2+z  =      8_455 Check
PLAT606_ALERT_4_G Solvent Accessible VOID(S) in Structure .......          ! Info
PLAT860_ALERT_3_G Number of Least-Squares Restraints ............        676 Note
PLAT869_ALERT_4_G ALERTS Related to the Use of SQUEEZE  Suppressed         ! Info
PLAT883_ALERT_1_G No Info/Value for _atom_sites_solution_primary .     Please Do !
PLAT909_ALERT_3_G Percentage of I>2sig(I) Data at Theta(Max) Still       53% Note
PLAT910_ALERT_3_G Missing # of FCF Reflection(s) Below Theta(Min).         2 Note
PLAT967_ALERT_5_G Note: Two-Theta Cutoff Value in Embedded .res ..      74.0 Degree
PLAT978_ALERT_2_G Number C-C Bonds with Positive Residual Density.         0 Info
```

---

1 **ALERT level A** = Most likely a serious problem - resolve or explain
14 **ALERT level B** = A potentially serious problem, consider carefully
38 **ALERT level C** = Check. Ensure it is not caused by an omission or oversight
60 **ALERT level G** = General information/check it is not something unexpected

6 ALERT type 1 CIF construction/syntax error, inconsistent or missing data
75 ALERT type 2 Indicator that the structure model may be wrong or deficient
13 ALERT type 3 Indicator that the structure quality may be low
18 ALERT type 4 Improvement, methodology, query or suggestion
1 ALERT type 5 Informative message, check

It is advisable to attempt to resolve as many as possible of the alerts in all categories. Often the minor alerts point to easily fixed oversights, errors and omissions in your CIF or refinement strategy, so attention to these fine details can be worthwhile. In order to resolve some of the more serious problems it may be necessary to carry out additional measurements or structure refinements. However, the purpose of your study may justify the reported deviations and the more serious of these should normally be commented upon in the discussion or experimental section of a paper or in the "special_details" fields of the CIF. checkCIF was carefully designed to identify outliers and unusual parameters, but every test has its limitations and alerts that are not important in a particular case may appear. Conversely, the absence of alerts does not guarantee there are no aspects of the results needing attention. It is up to the individual to critically assess their own results and, if necessary, seek expert advice.

Publication of your CIF in IUCr journals

A basic structural check has been run on your CIF. These basic checks will be run on all CIFs submitted for publication in IUCr journals (*Acta Crystallographica*, *Journal of Applied Crystallography*, *Journal of Synchrotron Radiation*); however, if you intend to submit to *Acta Crystallographica Section C* or *E* or *IUCrData*, you should make sure that full publication checks are run on the final version of your CIF prior to submission.

Publication of your CIF in other journals

Please refer to the *Notes for Authors* of the relevant journal for any special instructions relating to CIF submission.

PLATON version of 18/05/2022; check.def file version of 17/05/2022

Datablock y7-2_sq - ellipsoid plot

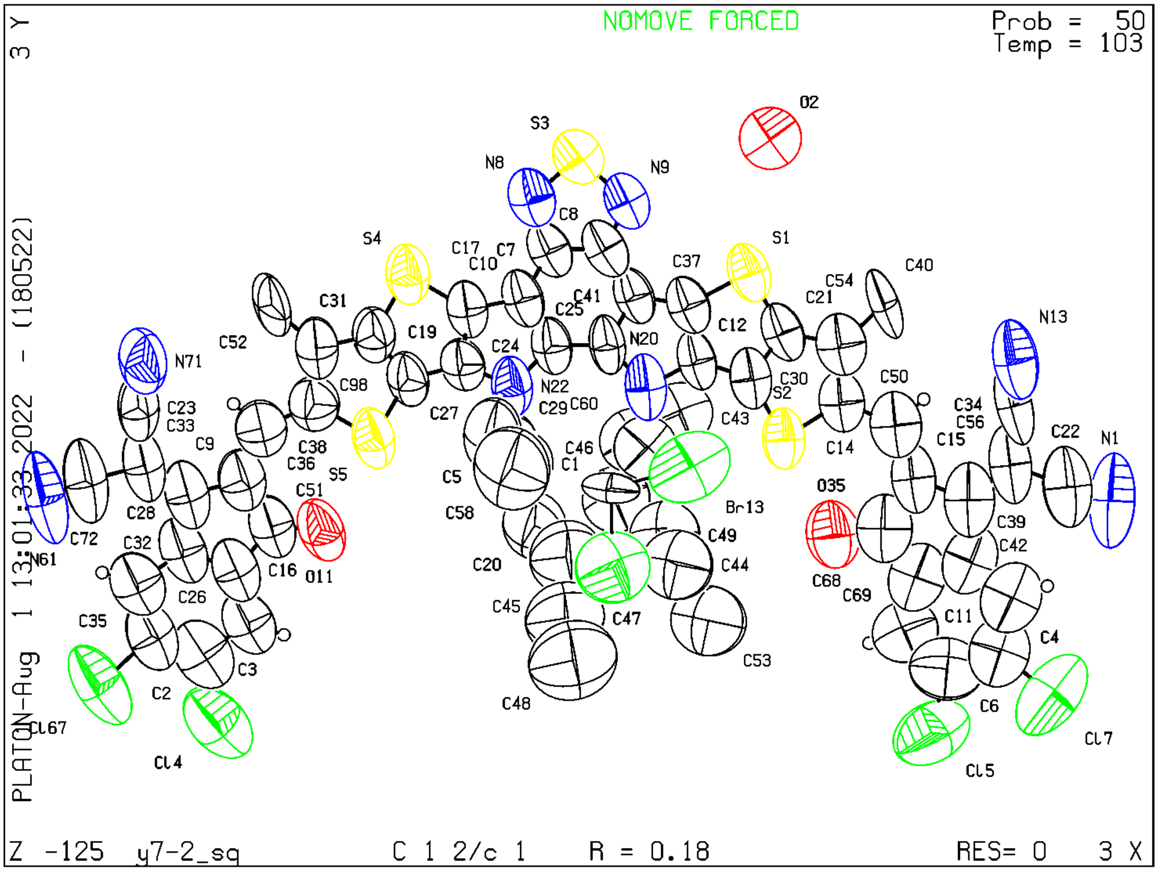

Supplement: Supplementary file 2 — Supporting Information [file ADVS-11-2307569-s002.zip › advs202307569-sup-0002-cif/BTIC-4Cl-checkcif.pdf]
